# A universal scaling relationship between body mass and proximal limb bone dimensions in quadrupedal terrestrial tetrapods

**DOI:** 10.1186/1741-7007-10-60

**Published:** 2012-07-10

**Authors:** Nicolás E Campione, David C Evans

**Affiliations:** 1 Department of Ecology and Evolutionary Biology, University of Toronto, 25 Willcocks Street, Toronto, Ontario, Canada M5S 3B2; 2 Department of Palaeobiology, Royal Ontario Museum, 100 Queen's Park, Toronto, Ontario, Canada M5S 2C6

## Abstract

**Background:**

Body size is intimately related to the physiology and ecology of an organism. Therefore, accurate and consistent body mass estimates are essential for inferring numerous aspects of paleobiology in extinct taxa, and investigating large-scale evolutionary and ecological patterns in the history of life. Scaling relationships between skeletal measurements and body mass in birds and mammals are commonly used to predict body mass in extinct members of these crown clades, but the applicability of these models for predicting mass in more distantly related stem taxa, such as non-avian dinosaurs and non-mammalian synapsids, has been criticized on biomechanical grounds. Here we test the major criticisms of scaling methods for estimating body mass using an extensive dataset of mammalian and non-avian reptilian species derived from individual skeletons with live weights.

**Results:**

Significant differences in the limb scaling of mammals and reptiles are noted in comparisons of limb proportions and limb length to body mass. Remarkably, however, the relationship between proximal (stylopodial) limb bone circumference and body mass is highly conserved in extant terrestrial mammals and reptiles, in spite of their disparate limb postures, gaits, and phylogenetic histories. As a result, we are able to conclusively reject the main criticisms of scaling methods that question the applicability of a universal scaling equation for estimating body mass in distantly related taxa.

**Conclusions:**

The conserved nature of the relationship between stylopodial circumference and body mass suggests that the minimum diaphyseal circumference of the major weight-bearing bones is only weakly influenced by the varied forces exerted on the limbs (that is, compression or torsion) and most strongly related to the mass of the animal. Our results, therefore, provide a much-needed, robust, phylogenetically corrected framework for accurate and consistent estimation of body mass in extinct terrestrial quadrupeds, which is important for a wide range of paleobiological studies (including growth rates, metabolism, and energetics) and meta-analyses of body size evolution.

## Background

In extant taxa, body size is recognized as one of the most important biological properties because it strongly correlates with numerous physiological and ecological factors, such as metabolic rate [1-3], growth rate [4,5], fecundity [6], diversity [7], and population density [8,9], as well as home range and land area [6,10,11], which are related to the productivity of the host environment [12]. Due to these relationships, estimates of body mass (the standard measure of body size) are essential for inferring the paleobiology of extinct taxa, and investigating large-scale evolutionary and ecological patterns in the history of life.

Due to the biological implications of body size, it is not surprising that numerous paleontological studies have used body mass estimates to reconstruct and interpret: patterns of body size evolution [13-22], brain-size allometry and evolution [23-26], the evolution of reproduction [27-29], growth rates [30,31], postural allometry and locomotion [14,32,33], metabolism [34-36], paleotemperature [37], visceral organ size [38], and community and trophic structures [10,39,40]. In order to infer these biological properties, studies require the use of an estimate or proxy of body size, which can have a large effect on the final interpretation. As a result, it is important to understand the set of assumptions/errors incurred by body size estimates and proxies.

Currently, there are two types of methods used to estimate body mass in extinct animals: volumetric reconstructions and skeletal scaling relationships. The latter method is commonly used to predict body mass in extinct members of relatively recent crown clades (that is, of Mesozoic origin) such as Mammalia and Aves [21,41-45]. However, in stem groups (for example, non-avian dinosaurs and non-mammalian synapsids), estimations are often based on volumetric reconstructions, which involve physical three-dimensional scale models [46,47], graphic double integration of two-dimensional reconstructions [48-50], or computer-generated life reconstructions [51-55]. Such estimates are widely used in the literature (for example, [35,38]) despite the fact that they are prone to a considerable amount of error. In a typical example, body mass estimates for a single mounted skeleton of *Brachiosaurus brancai *recently published by the same research group have resulted in estimates of 38 tonnes and 74.4 tonnes [54,56]. Such differences in estimates are the result of differing interpretations of a multitude of factors associated with the mass and proportion of an organism's tissues and organs [57], or, perhaps most importantly, the effects of air sacs and lungs, which will likely have a large effect on specific gravity (the total body density of the animal in relation to water), needed to estimate mass from a volume. Within non-avian reptiles specific gravity has been noted to range from 0.8 to 1.2 [46,48]; however, given the varying levels of bone pneumaticity observed in saurischian dinosaurs [58,59], and the fact that birds typically exhibit lower densities than mammals and other reptiles [60], it is almost certain that the specific gravity of extinct animals also varied [59]. As a result, assumptions based on a set density parameter will considerably affect a mass estimate [54,56]. Perhaps more importantly, the numerous assumptions about soft tissue properties and body shape (for example, muscle sizes) in many of the models make it difficult to control for sources of error and to determine the confidence associated with a given mass estimate, although recent computational modelling advances attempt to outline maximum and minimum body mass bounds (for example, [54,61,62]). Despite the complications associated with life reconstructions of extinct taxa, models are important for testing numerous biomechanical hypotheses [61,63-68]. Therefore, it is important that models be constrained by data derived from extant taxa, such as those obtained from scaling relationships.

An alternative method to reconstructions, and one that can be used to test and constrain scale and computational models ([55]), is the use of scaling relationships between body mass and skeletal dimensions derived from extant taxa. A skeletal measure, if strongly related to body mass, will provide an estimate that controls for the sources of error associated with making a reconstruction, such as determination of tissue volume and specific gravity, which are virtually impossible to constrain in life-reconstructions. Furthermore, skeletal measurements are generally easier to obtain than full body scale reconstructions, especially for taxa that are only partially preserved, and are therefore more practical estimators in large-scale evolutionary and ecological studies (for example, [15-17,20]). Finally, the variation in the extant dataset can be used to quantify the degree of confidence in the estimated parameter, and can thus provide a range in which a particular body mass is likely to fall, thereby providing a constraint for estimates produced by reconstructed models. Scaling methods are almost universally accepted as a means to estimate body mass accurately for extinct taxa of crown groups, such as mammals and birds (for example, [17,42]), but have been extensively criticized when applied to more distantly related stem taxa that fall outside the body size range observable in extant representatives, such as *Indricotherium *[69], xenarthrans [43], and non-avian dinosaurs [70-72]. For the first two groups, studies have since shown that scaling relationships still provide the most reliable mass estimates [43,69].

Dinosaurian body masses are still generally estimated using reconstructions, with the exception of two studies [45,73]. The pioneering work completed by Anderson *et al. *[73], herein referred to as the Anderson method, suggested that the body mass of dinosaurs could be estimated using the measured scaling relationship between live mass and total circumference of the stylopodia (humerus + femur) derived from a sample of 33 species of extant terrestrial mammals. Although the Anderson method provides a more objective way to estimate body mass in extinct taxa, it has been criticized by numerous authors (for example, [49,56,61,70,71,74-76]). Here we use an extensive dataset of extant mammals and non-avian reptiles compiled from individual skeletons of live-weighed animals, in order to directly test the three main criticisms made towards the use of a universal limb scaling relationship to estimate body mass in extinct terrestrial amniotes:

1. The widely cited Anderson method, especially among non-avian dinosaur researchers, is criticized based on its use of a taxonomically biased sample towards ungulates (for example, [70]). Studies examining limb-scaling patterns in mammals have noted that the limb proportions of ungulates differ from those of other mammals [70,77,78]. However, whether ungulates differ from other groups of mammals in their scaling patterns of limb circumference to body mass has not been directly tested.

2. Differences in gait and limb posture impart different stress regimes on the limbs [79,80]. These differences may affect limb morphology, thereby negating the applicability of a single equation to estimate body mass in a variety of extinct vertebrates. Given different stress regimes, we test for differential limb scaling between animals of various gaits and limb posture by comparing differently sized sub-samples of mammals, and parasagittal mammals to sprawling reptiles.

3. Residual outliers (large residual values) and extreme outliers (values at the upper and lower extremes of the dataset) can have a large effect on regression coefficients [81]. The problem of residual outliers in the large-bodied mammalian sample of Anderson *et al. *[73] was discussed by Packard *et al. *[82]. We have expanded the sample size of the large-bodied dataset and will address the effect that potential residual outliers have on the circumference to body mass relationship. The effect of extreme outliers on limb scaling is, in part, mediated by logarithmic transformation of the data, but will also be assessed through size class comparisons. Although the issue of body mass extrapolation to giant extinct taxa (for example, Sauropoda; [50,72]) will always exist, the vast majority of extinct animals, including most non-avian dinosaurs, fall within the body mass range of extant taxa.

All three of these criticisms are tested for the first time, within the context of 200 mammal and 47 non-avian reptile species [See Additional file 1, Dataset]. Based on our results we develop a universal scaling equation between the total circumference of the stylopodia and body mass that is applicable to all terrestrial quadrupeds, and permits estimation of body mass in extinct taxa along with an error factor that can constrain estimates for use in future paleobiological studies.

## Results

### Raw data results

**Figure 1:**
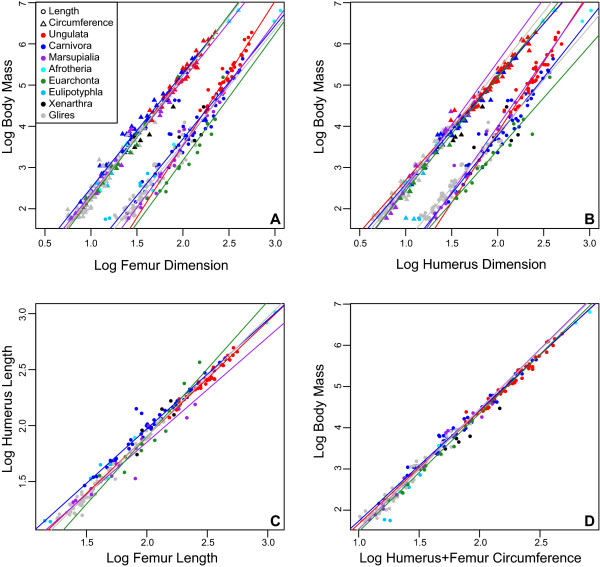
**Limb scaling patterns in mammalian clades**. Lines are fitted based on the SMA results presented in Table 1. (**A**) Log femoral length and circumference plotted against log body mass. (**B**) Log humeral length and circumference against log body mass. (**C**) Log femoral length plotted against log humeral length. (**D**) The log of combined humeral and femoral circumference against log body mass. SMA, standardized major axis.

**Figure 2:**
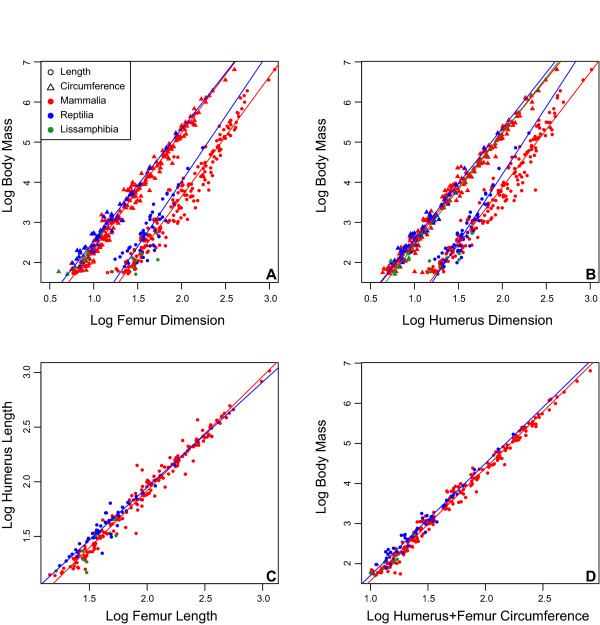
**Limb scaling patterns in quadrupedal terrestrial tetrapods**. Lines are fitted based on the SMA results presented in Table 1. Lissamphibians are plotted (green) but no line was fitted due to its small sample size and body mass range. (**A**) Log femoral length and circumference plotted against log body mass. (**B**) Log humeral length and circumference against log body mass. (**C**) Log femoral length plotted against log humeral length. (**D**) The log combined humeral and femoral circumference against log body mass. SMA, standardized major axis.

**Figure 3:**
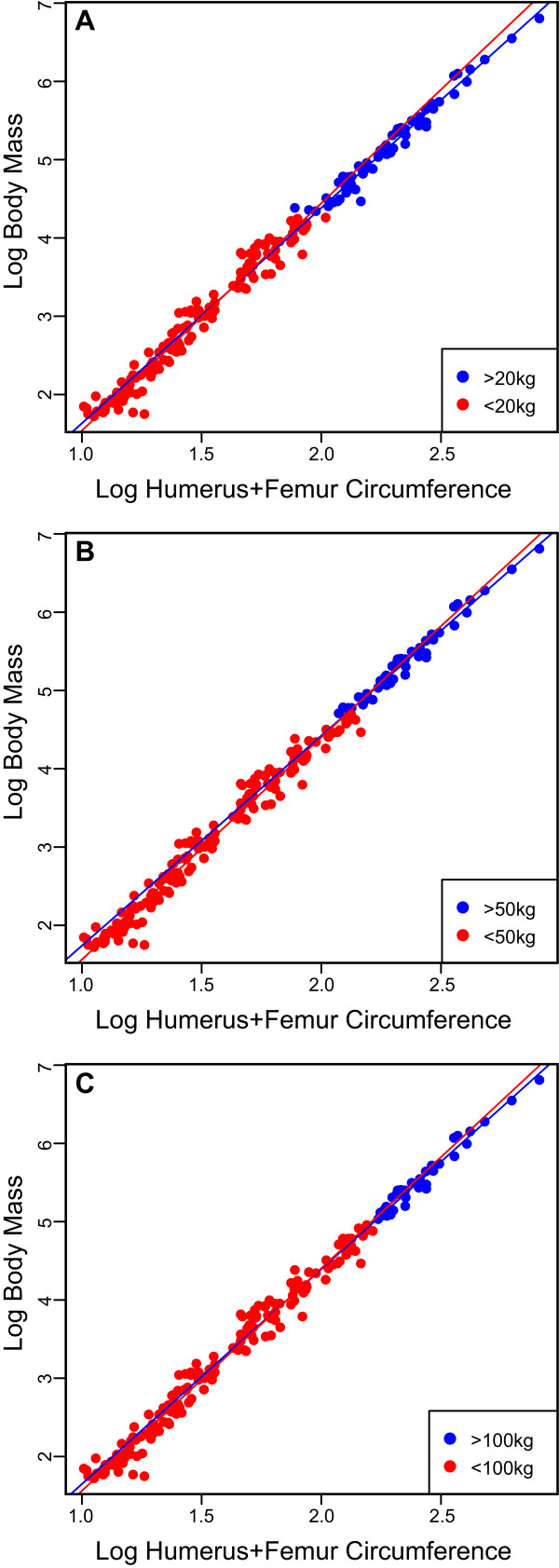
**Limb scaling patterns in different mammalian size classes**. Lines are fitted based on the SMA results presented in Table 4. All three comparisons plot the log total stylopodial circumference against log body mass in the mammalian sample of the dataset. Size class comparisons are based on previously studied thresholds discussed in the text [78,93,94]. Mammals above and below 20 kg (**A**), 50 kg (**B**), and 100 kg (**C**). SMA, standardized major axis.

Results from the standardized major axis (SMA) analyses comparing clades based on the raw non-phylogenetically corrected data are provided in Figures 1 and 2, and Table 1; comparisons are summarized in Tables 2 and 3. Size class comparisons are presented in Figure 3 and Tables 4 and 5. All analyses show strong correlations with each other, and to body mass (that is, size) as indicated by a mean coefficient of determination of 0.9446 ± 0.0093 for the clade comparisons, and 0.914 ± 0.014 for comparisons between size classes.

**Table 1:** Stylopodial scaling in mammals and non-avian reptiles.

Analysis (*x *vs. *y*)	Sample	N	*m*	*m *95% CI	*b*	95% +CI	R^2^	**Sim**.
L_F _vs. C_F_	All	234	1.0301	1.0616 to 0.9996	-0.6020	-0.542 to -0.6619	0.9459	G
	Mammalia	188	1.0332	1.0677 to 0.9999	-0.6148	-0.5469 to -0.6827	0.9484	G
	Reptilia	46	1.1751	1.3184 to 1.0474	-0.8115	-0.5884 to -1.0347	0.8560	> G, < E
	Ungulata	32	1.2014	1.3338 to 1.0821	-0.9810	-0.6676 to -1.2943	0.9211	> G, < E
	Carnivora	46	0.9840	1.0888 to 0.8893	-0.5409	-0.3317 to -0.75	0.8887	G
	Marsupialia	14	1.0774	1.1467 to 1.0123	-0.7317	-0.6057 to -0.8577	0.9902	> G, < E
	Euarchonta	14	1.0251	1.2141 to 0.8656	-0.7382	-0.3835 to -1.0929	0.9269	G
	Glires	66	0.9542	1.0334 to 0.8811	-0.4716	-0.3454 to -0.5979	0.8978	G

L_H _vs. C_H_	All	234	1.0644	1.0971 to 1.0326	-0.6229	-0.5626 to -0.6831	0.9452	> G, < E
	Mammalia	187	1.0603	1.0967 to 1.0252	-0.6199	-0.5511 to -0.6887	0.9459	> G, < E
	Reptilia	47	1.2190	1.3355 to 1.1126	-0.8536	-0.6724 to -1.0348	0.9072	> G, < E
	Ungulata	32	1.3083	1.4325 to 1.1949	-1.1391	-0.8529 to -1.4254	0.9407	> G, < E
	Carnivora	46	1.0814	1.1777 to 0.9929	-0.7101	-0.5193 to -0.9009	0.9209	G
	Marsupialia	14	1.0472	1.187 to 0.9238	-0.6059	-0.3774 to -0.8343	0.9601	G
	Euarchonta	14	0.9175	1.0816 to 0.7782	-0.4785	-0.1826 to -0.7744	0.9309	G
	Glires	66	0.9296	0.9931 to 0.8702	-0.4116	-0.3166 to -0.5066	0.9300	G

L_F _vs. BM	All	234	2.9307	3.0323 to 2.8325	-2.1677	-1.9744 to -2.3611	0.9306	G
	Mammalia	188	2.9930	3.0974 to 2.8922	-2.3410	-2.1359 to -2.5461	0.9439	G
	Reptilia	46	3.2500	3.7486 to 2.8177	-2.4800	-1.7132 to -3.2468	0.7778	G
	Ungulata	32	3.4979	3.8785 to 3.1545	-3.4591	-2.5578 to -4.3603	0.9230	> G, < E
	Carnivora	46	2.7472	3.0791 to 2.451	-1.8012	-1.1427 to -2.4597	0.8584	G
	Marsupialia	14	2.9980	3.4286 to 2.6215	-2.4690	-1.7121 to -3.2258	0.9542	G
	Euarchonta	14	3.0622	3.7695 to 2.4877	-2.9486	-1.6443 to -4.253	0.8893	G
	Glires	66	2.7702	2.9779 to 2.5769	-1.9621	-1.6297 to -2.2944	0.9160	0

C_F _vs. BM	All	247	2.8479	2.8997 to 2.7969	-0.4587	-0.3845 to -0.5328	0.9794	< G, > E
	Mammalia	200	2.8977	2.9504 to 2.8459	-0.5615	-0.4829 to -0.64	0.9834	< G, > E
	Reptilia	47	2.7943	2.9801 to 2.6201	-0.2653	-0.057 to -0.4736	0.9540	E
	Ungulata	41	2.9204	3.1192 to 2.7344	-0.6173	-0.232 to -1.0027	0.9586	G
	Carnivora	48	2.7893	2.9182 to 2.6661	-0.2895	-0.0946 to -0.4844	0.9768	E
	Marsupialia	14	2.7827	3.1222 to 2.4801	-0.4328	-0.0138 to -0.8518	0.9664	G, E, S
	Euarchonta	15	2.9728	3.1874 to 2.7727	-0.7271	-0.4393 to -1.0149	0.9864	G
	Glires	66	2.9031	3.084 to 2.7328	-0.5929	-0.3965 to -0.7893	0.9413	G

L_H _vs. BM	All	234	2.8653	2.9489 to 2.7841	-1.8284	-1.6745 to -1.9823	0.9506	0
	Mammalia	187	2.8626	2.9522 to 2.7756	-1.8476	-1.6778 to -2.0175	0.9548	0
	Reptilia	47	3.3718	3.704 to 3.0694	-2.5472	-2.0315 to -3.0629	0.9018	> G, < E
	Ungulata	32	3.4092	3.8036 to 3.0558	-2.9639	-2.0630 to -3.8648	0.9135	> G, < E
	Carnivora	46	2.8202	3.0641 to 2.5957	-1.8667	-1.3831 to -2.3503	0.9253	G
	Marsupialia	14	3.1988	3.6508 to 2.8027	-2.3972	-1.6611 to -3.1333	0.9556	G
	Euarchonta	14	2.5359	2.9736 to 2.1627	-1.6484	-0.8577 to -2.4391	0.9354	0
	Glires	66	2.6071	2.766 to 2.4573	-1.3946	-1.1559 to -1.6332	0.9438	0

C_H _vs. BM	All	247	2.6861	2.7322 to 2.6406	-0.1438	-0.0788 to -0.2087	0.9816	E
	Mammalia	200	2.6938	2.7445 to 2.6439	-0.1655	-0.0913 to -0.2398	0.9823	E
	Reptilia	47	2.7661	2.9296 to 2.6117	-0.1862	-0.0049 to -0.3675	0.9634	E
	Ungulata	41	2.5273	2.7222 to 2.3464	0.1672	0.544 to -0.2097	0.9473	E
	Carnivora	48	2.5959	2.7027 to 2.4933	-0.0012	0.1613 to -0.1637	0.9815	E, S
	Marsupialia	14	3.0547	3.4219 to 2.7269	-0.5465	-0.1208 to -0.9721	0.9673	G
	Euarchonta	15	2.7558	2.9725 to 2.5548	-0.3168	-0.0345 to -0.5992	0.9840	E
	Glires	66	2.8045	2.9447 to 2.671	-0.2403	-0.0986 to -0.3819	0.9618	< G, > E

L_F _vs. L_H_	All	233	1.0246	1.0469 to 1.0027	-0.1206	-0.0778 to -0.1634	0.9723	-
	Mammalia	187	1.0450	1.0682 to 1.0223	-0.1707	-0.1248 to -0.2166	0.9771	-
	Reptilia	46	0.9644	1.0644 to 0.8739	0.0190	0.176 to -0.1379	0.8943	-
	Ungulata	32	1.0260	1.107 to 0.9509	-0.1452	0.0491 to -0.3395	0.9584	-
	Carnivora	46	0.9741	1.0283 to 0.9227	0.0232	0.1338 to -0.0874	0.9682	-
	Marsupialia	14	0.9372	1.1182 to 0.7856	-0.0224	0.2894 to -0.3344	0.9204	-
	Euarchonta	14	1.2075	1.4238 to 1.0241	-0.5127	-0.106 to -0.9195	0.9307	-
	Glires	66	1.0625	1.1019 to 1.0246	-0.2177	-0.1536 to -0.2818	0.9788	-

C_H+F _vs. BM	All	247	2.7779	2.8191 to 2.7374	-1.1564	-1.086 to -1.2267	0.9863	-
	Mammalia	200	2.8071	2.8495 to 2.7654	-1.2289	-1.1541 to -1.3037	0.9886	-
	Reptilia	47	2.7933	2.9496 to 2.6452	-1.0833	-0.8636 to -1.3031	0.9671	-
	Ungulata	41	2.7319	2.8959 to 2.5773	-1.0660	-0.6989 to -1.4331	0.9676	-
	Carnivora	48	2.6921	2.7969 to 2.5911	-0.9568	-0.7669 to -1.1466	0.9834	-
	Marsupialia	14	2.9125	3.1855 to 2.6628	-1.3738	-0.9658 to -1.7817	0.9797	-
	Euarchonta	15	2.8692	3.0561 to 2.6937	-1.3928	-1.0911 to -1.6946	0.9889	-
	Glires	66	2.8850	3.0113 to 2.764	-1.3260	-1.1561 to -1.4960	0.9705	-

Standardized Major Axis equation shown in the format *y = mx + b*. The particular theoretical scaling model (Sim.) followed by the slope is represented by G, geometric similarity, E, elastic similarity, or S, static similarity. Scaling patterns that fall between models are represented by > or <, and those that do not follow any pattern (that is, above or below all predicted models) are represented by a 0. BM, body mass; C_F_, femoral circumference; C_H_, humeral circumference; C_H+F_, total humeral and femoral circumference; CI, confidence interval; L_F_, femoral length; L_H_, humeral length.

**Table 2:** Slope and intercept comparisons of stylopodial scaling patterns in mammalian clades.

		*m *95% CI				*m *LRT				*b *95% CI				*b *t-test				*b' *t-test			
		
		Marsupialia	Glires	Euarchonta	Ungulata	Marsupialia	Glires	Euarchonta	Ungulata	Marsupialia	Glires	Euarchonta	Ungulata	Marsupialia	Glires	Euarchonta	Ungulata	Marsupialia	Glires	Euarchonta	Ungulata
L_F _vs. C_F_	Carnivora								*												
	
	Ungulata		*		-		*		-		*		-				-				-
	
	Euarchonta			-	-			-	-			-	-			-	-			-	-
	
	Glires		-	-	-	°	-	-	-	*	-	-	-		-	-	-		-	-	-

L_H _vs. C_H_	Carnivora				*		*		**		*				*		*				
	
	Ungulata	*	*	*	-	*	**	**	-	*	*	*	-	*	**	*	-				-
	
	Euarchonta			-	-			-	-			-	-			-	-			-	-
	
	Glires			-	-		-	-	-		-	-	-		-	-	-		-	-	-

L_F _vs. BM	Carnivora				*				*				*								
	
	Ungulata		*		-		**		-		*		-				-				-
	
	Euarchonta			-	-			-	-			-	-			-	-			-	-
	
	Glires		-	-	-		-	-	-		-	-	-		-	-	-		-	-	-

C_F _vs. BM	Carnivora																				
	
	Ungulata				-				-				-				-				-
	
	Euarchonta			-	-			-	-			-	-			-	-			-	-
	
	Glires		-	-	-		-	-	-		-	-	-		-	-	-		-	-	-

L_H _vs. BM	Carnivora								*												
	
	Ungulata		*	*	-		**	*	-		*		-		*		-				-
	
	Euarchonta			-	-	*		-	-			-	-			-	-			-	-
	
	Glires	*	-	-	-	*	-	-	-	*	-	-	-	*	-	-	-		-	-	-

C_H _vs. BM	Carnivora	*				°	°							*							
	
	Ungulata	*			-	°	°		-				-	°			-				-
	
	Euarchonta			-	-			-	-			-	-			-	-			-	-
	
	Glires		-	-	-		-	-	-		-	-	-		-	-	-		-	-	-

L_F _vs. L_H_	Carnivora										*	*			**	**					
	
	Ungulata				-				-				-				-				-
	
	Euarchonta			-	-			-	-			-	-	°	°	-	-			-	-
	
	Glires		-	-	-		-	-	-		-	-	-	°	-	-	-		-	-	-

C_H+F _vs. BM	Carnivora										*				°						
	
	Ungulata				-				-				-				-				-
	
	Euarchonta			-	-			-	-			-	-			-	-			-	-
	
	Glires		-	-	-		-	-	-		-	-	-		-	-	-		-	-	-

Standardized major axis equation shown in the format *y = mx + b*. Symbols: (°) represents differences at 90 to 95% (0.1 <*P *> 0.05); (*) at 95-99% (0.05 <*P *> 0.01); and (**) at greater than 95% (*P *< 0.01). Otherwise, *P*-values are > 0.1. All *P*-values are adjusted for multiple comparisons using FDR. Hyphens (-) represent duplicate comparisons. Significant differences using 95% CI are assessed on whether the intervals overlap or not; non-overlapping comparisons are indicated with an asterisk (*). 95% CI, comparisons based on 95% confidence intervals; *b'*, intercept adjusted to correspond to the minimum value along the x-axis; BM, body mass; C_F_, femoral circumference; C_H_, humeral circumference; C_H+F_, total humeral and femoral circumference; FDR, false discovery rate; L_F_, femoral length; L_H_, humeral length; LRT, comparisons based on a likelihood ratio test (slope only); t-test, comparisons based on a two-tailed t-test (intercept only).

**Table 3:** Slope and intercept comparisons of stylopodial scaling patterns in mammals and non-avian reptiles.

	All Data					Mammals < 168 kg^a^				
	***m*CI**	***m*P**	***b*CI**	***b*P**	***b'*P**	***m*CI**	***m*P**	***b*CI**	***b*P**	***b'*P**

L_F _vs C_F_		°				*	°			

L_H _vs C_H_	*	*				*	**		°	

L_F _vs BM										

C_F _vs BM			*							

L_H _vs BM	*	**	*	°		*	**		*	

C_H _vs BM										

L_F _vs L_H_				°					*	

C_H+F _vs BM										

Standardized major axis equation shown in the format *y = mx + b*. Symbols: (°) represents differences at 90 to 95% (0.1 <*P *> 0.05); (*) at 9 to 99% (0.05 <*P *> 0.01); and (**) at greater than 95% (*P *< 0.01). Otherwise, *P*-values are > 0.1. All *P*-values are adjusted for multiple comparisons using FDR. Significant differences using 95% CI are assessed on whether the intervals overlap or not; non-overlapping comparisons are indicated with an asterisk (*). ^a^comparisons based on a subset of the mammalian dataset that has the same body mass range as the total reptilian dataset [See Additional file 2, Table S1]; *m*CI, slope comparisons based on 95% confidence intervals; *m*P, slope comparisons based on likelihood ratio test; *b*CI, intercept comparisons based 95% confidence intervals; *b*P, intercept comparisons based on two-tailed t-test; *b'*P, t-test comparison of adjusted intercepts to the minimum value along the x-axis; BM, body mass; C_F_, femoral circumference; FDR, false discovery rate; L_H_, humeral length; C_H_, humeral circumference; C_H+F_, total humeral and femoral circumference; L_F_, femoral length.

**Table 4:** Stylopodial scaling in mammals of different sizes.

Analysis (*x *vs. *y*)	Sample	N	*m*	*m *95% CI	*b*	*b *to 95% to CI	R^2^	Sim
L_F _vs. C_F_	< 20 kg	136	0.8868	0.9335 to 0.8424	-0.3733	-0.2921 to -0.4545	0.9095	0
	> 20 kg	52	1.0000	1.1370 to 0.8795	-0.4907	-0.1714 to -0.8100	0.7945	G
	< 50 kg	150	0.9486	0.9987 to 0.9009	-0.4715	-0.3819 to -0.5611	0.8993	G
	> 50 kg	38	1.1331	1.2731 to 1.0084	-0.8317	-0.4935 to -1.1699	0.8806	> G, < E
	< 100 kg	158	0.9659	1.0123 to 0.9216	-0.5000	-0.4155 to -0.5845	0.9119	G
	> 100 kg	30	1.1059	1.2659 to 0.9661	-0.7572	-0.3679 to -1.1465	0.8774	G

L_H _vs. C_H_	< 20 kg	135	0.8778	0.9248 to 0.8331	-0.3345	-0.2567 to -0.4124	0.9073	0
	> 20 kg	52	1.1541	1.2900 to 1.0326	-0.7954	-0.4848 to -1.1060	0.8459	> G, < E
	< 50 kg	149	0.9040	0.9990 to 0.9040	-0.4445	-0.3613 to -0.5277	0.9060	0
	> 50 kg	38	1.1856	1.3524 to 1.0394	-0.8774	-0.4879 to -1.2668	0.8475	> G, < E
	< 100 kg	157	0.9710	1.0166 to 0.9274	-0.4764	-0.3967 to -0.5560	0.9161	G
	> 100 kg	30	1.1229	1.3132 to 0.9602	-0.7114	-0.2646 to -1.1582	0.8352	G

L_F _vs. BM	< 20 kg	136	2.6288	2.7825 to 2.4836	-1.7421	-1.4756 to -2.0086	0.8892	0
	> 20 kg	52	2.8571	3.2511 to 2.5108	-1.8964	-0.9788 to -2.8141	0.7920	G
	< 50 kg	150	2.7619	2.9166 to 2.6754	-1.9510	-1.6751 to -2.2270	0.8873	0
	> 50 kg	38	3.1523	3.5377 to 2.8089	-2.6399	-1.7089 to -3.5709	0.8831	G
	< 100 kg	158	2.8104	2.9526 to 2.6750	-2.0305	-1.7717 to -2.2893	0.9025	0
	> 100 kg	30	3.0497	3.5022 to 2.6557	-2.3587	-1.2593 to -3.4582	0.8715	G

C_F _vs. BM	< 20 kg	138	2.9559	3.0735 to 2.8429	-0.6266	-0.4858 to -0.7675	0.9471	G
	> 20 kg	62	2.8638	3.0353 to 2.7020	-0.5040	-0.1723 to -0.8358	0.9492	G
	< 50 kg	153	2.9054	3.0013 to 2.8126	-0.5716	-0.4504 to -0.6928	0.9592	G
	> 50 kg	47	2.7816	2.9651 to 2.6094	-0.3222	0.0436 to -0.6880	0.9546	E
	< 100 kg	164	2.9117	2.9945 to 2.8312	-0.5784	-0.4698 to -0.6870	0.9674	< G, > E
	> 100 kg	36	2.7946	3.0538 to 2.5575	-0.3497	0.1751 to -0.8745	0.9351	G, E

L_H _vs. BM	< 20 kg	135	2.4386	2.5604 to 2.3225	-1.1878	-0.9858 to -1.3898	0.9192	0
	> 20 kg	52	2.9807	3.2978 to 2.6940	-2.0078	-1.2791 to -2.7365	0.8728	G
	< 50 kg	149	2.5866	2.7051 to 2.4734	-1.4091	-1.2063 to -1.6120	0.9245	0
	> 50 kg	38	3.0525	3.4941 to 2.6667	-2.1794	-1.1501 to -3.2088	0.8392	G
	< 100 kg	157	2.6465	2.7582 to 2.5394	-1.5013	-1.3060 to -1.6966	0.9321	0
	> 100 kg	30	2.9405	3.4742 to 2.4888	-1.8798	-0.6326 to -3.1269	0.8127	G

C_H _vs. BM	< 20 kg	138	2.7768	2.8898 to 2.6683	-0.2550	-0.1255 to -0.3845	0.9447	E
	> 20 kg	62	2.5793	2.7425 to 2.4258	0.0509	0.3671 to -0.2653	0.9434	E, S
	< 50 kg	153	2.7188	2.8130 to 2.6277	-0.1941	-0.0793 to -0.3088	0.9551	E
	> 50 kg	47	2.5612	2.7123 to 2.4184	0.1031	0.4070 to -0.2008	0.9635	E, S
	< 100 kg	164	2.7253	2.8067 to 2.6463	-0.2005	-0.0972 to -0.3038	0.9640	E
	> 100 kg	36	2.6488	2.8634 to 2.4504	-0.0887	0.3518 to -0.5293	0.9500	E, S

L_F _vs. L_H_	< 20 kg	135	1.0776	1.1143 to 1.0422	-0.2261	-0.1618 to -0.2904	0.9619	-
	> 20 kg	52	0.9586	1.0632 to 0.8642	0.0374	0.2839 to -0.2092	0.8666	-
	< 50 kg	149	1.0672	1.1041 to 1.0315	-0.2079	-0.1414 to -0.2744	0.9564	-
	> 50 kg	38	1.0327	1.1337 to 0.9407	-0.1509	0.0956 to -0.3973	0.9236	-
	< 100 kg	157	1.0613	1.0945 to 1.0291	-0.1983	-0.1374 to -0.2592	0.9624	-
	> 100 kg	30	1.0371	1.1743 to 0.9160	-0.1629	0.1727 to -0.4984	0.8965	-

C_H+F _vs. BM	< 20 kg	138	2.9032	2.9989 to 2.8105	-1.3628	-1.2223 to -1.5032	0.9634	-
	> 20 kg	62	2.7519	2.8828 to 2.6269	-1.1186	-0.8251 to -1.4120	0.9674	-
	< 50 kg	153	2.8383	2.9165 to 2.7622	-1.2743	-1.1542 to -1.3945	0.9714	-
	> 50 kg	47	2.6819	2.8173 to 2.5530	-0.9409	-0.6286 to -1.2531	0.9731	-
	< 100 kg	164	2.8409	2.9084 to 2.7750	-1.2778	-1.1709 to 1.3846	0.9771	-
	> 100 kg	36	2.7442	2.9343 to 2.5663	-1.0954	-0.6491 to -1.5416	0.9630	-

Standardized Major Axis equation shown in the format *y = mx + b*. The particular theoretical scaling model (Sim.) followed by the slope is represented by G, geometric similarity, E, elastic similarity, or S, static similarity. Scaling patterns that fall between models are represented by > or <, and those that do not follow any pattern (that is, above or below all predicted models) are represented by a 0. BM, body mass; C_F_, femoral circumference; C_H_, humeral circumference; C_H+F_, total humeral and femoral circumference; L_F_, femoral length; L_H_, humeral length

**Table 5:** Slope and intercept comparisons of stylopodial scaling patterns in different mammalian size classes.

	20 kg					50 kg					100 kg				
	***m*CI**	***m*P**	***b*CI**	***b*P**	***b'*P**	***m*CI**	***m*P**	***b*CI**	***b*P**	***b'*P**	***m*CI**	***m*P**	***b*CI**	***b*P**	***b'*P**

L_F _vs C_F_						*	*								

L_H _vs C_H_	*	**	*	*		*	**								

L_F _vs BM															

C_F _vs BM															

L_H _vs BM	*	**					*								

C_H _vs BM															

L_F _vs L_H_				°											

C_H+F _vs BM															

Standardized major axis equation shown in the format *y = mx + b*. Symbols: (°) represents differences at 90 to 95% (0.1 <*P *> 0.05); (*) at 9 to 99% (0.05 <*P *> 0.01); and (**) at greater than 95% (*P *< 0.01). Otherwise, *P*-values are > 0.1. All *P*-values are adjusted for multiple comparisons using FDR. All *P*-values are adjusted for multiple for multiple comparisons using FDR. Hyphens (-) represent duplicate comparisons. Significant differences using 95% CI are assessed on whether the intervals overlap or not; non-overlapping comparisons are indicated with an asterisk (*). *m*CI, slope comparisons based on 95% confidence intervals; *m*P, slope comparisons based on likelihood ratio test; *b*CI, intercept comparisons based 95% confidence intervals; *b*P, intercept comparisons based on two-tailed t-test; *b'*P, t-test comparison of adjusted intercepts to the minimum value along the x-axis; BM, body mass; C_F_, femoral circumference; FDR, false discovery rate; L_H_, humeral length; C_H_, humeral circumference; C_H+F_, total humeral and femoral circumference; L_F_, femoral length;*m*CI, slope comparisons based on 95% confidence intervals; *m*P, slope comparisons based on likelihood ratio test; *b*CI, intercept comparisons based 95% confidence intervals; *b*P, intercept comparisons based on two-tailed t-test; *b'*P, t-test comparison of adjusted intercepts to the minimum value along the x-axis; BM, body mass; C_H_, humeral circumference; C_H+F_, total humeral and femoral circumference; L_C_, femoral circumference;

L_F_, femoral length; L_H_, humeral length.

In total, 80 pairwise comparisons are made between mammalian clades (Tables 1 and 2). Of these comparisons, the 95% confidence intervals indicate 12 significant differences between scaling coefficients and 13 significant differences between intercepts. In comparison, the likelihood ratio test, the results of which are adjusted for multiple comparisons using the false discovery rate (FDR), reveals 14 significant differences between slopes, and a t-test of the true intercepts indicates ten significant differences; however, when the intercept is corrected and compared at a more biologically meaningful value, the minimum value along the x-axis, the t-test indicates that there are no significant differences in intercept.

Regardless of the comparison method used, the most significant variation is noted in the scaling of stylopodial proportions (length to circumference) of the humerus and femur, as well as in the scaling of humeral and femoral lengths with body mass (Figure 1; Tables 1 and 2). This is especially true for ungulates, which possess stylopodial proportions and lengths that scale significantly different from all other groups examined here. No significant differences in scaling coefficients were recovered in the scaling of either the humeral or femoral circumference to body mass using the likelihood ratio test, and only two differences were recovered by the 95% confidence interval comparisons in the scaling of humerus circumference to body mass (Marsupialia scales significantly higher than Ungulata and Carnivora).

In total, ten and 13 significant differences were noted in comparisons between intercepts using confidence intervals and a t-test, respectively, including a significant difference in the intercept of Carnivora and Glires using 95% confidence intervals in the comparison of total stylopodial (humerus + femur) circumference and body mass. However, visual inspection reveals major overlap between the data points at the minimum values along the x-axis (Figure 1) suggesting that significant differences may be due to extrapolation of the SMA line to a value of x = 0. This is likely a valid interpretation as an adjusted t-test comparing the intercepts at the minimum values along the x-axis (Table 2) indicates that intercepts are not significantly different between mammalian groups in any of the comparisons made here.

Mammalian and reptilian scaling patterns show similar scaling coefficients, overall. Of the eight comparisons, two scaling coefficients showed significant differences using both the 95% confidence intervals and the likelihood ratio test. More specifically, the humeral proportions and humeral length to body mass in reptiles scale above that observed for mammals (Figure 2; Tables 1 and 3). Comparison of the confidence intervals revealed significant differences in the intercepts of mammals and reptiles in the relationship between femur circumference and body mass, as well as humerus length to body mass. However, these differences were not recovered by either t-test. When the circumference of the humerus and femur is combined, all tests indicate that the total stylopodial circumference to body mass relationship of reptiles is statistically indifferentiable from that of mammals.

Finally, in order to assure that the results obtained for mammals and reptiles are not influenced by differences in body size range in the two samples, we re-ran the analyses using a subset of the mammalian dataset (N = 174), which corresponds to all mammals equal to, or below, the mass of the *Alligator mississippiensis *specimen (168 kg), the largest reptile measured in this study. In general, results of this pruned analysis were similar to those obtained with the entire mammalian dataset (Table 3)[See Additional file 2, Table S1]. In particular, comparisons of slopes based on the likelihood ratio test are identical. Differences between the two analyses were noted in comparisons using the 95% confidence intervals in which the pruned analysis revealed an additional difference in the scaling of femoral length and circumference between mammals and reptiles, but failed to recover a significant difference between intercepts in the scaling of femoral circumference to body mass. The t-test on the pruned data also revealed an additional difference between the intercepts of mammals and reptiles in the relationship of humeral length to body mass as well as femoral to humeral length. Despite differences in the scaling of stylopodial length, no significant differences were noted in the scaling of stylopodial circumference to body mass between mammals and reptiles.

Size class comparisons, based on the mammalian dataset (N = 200), at three different thresholds reveal greater variation in scaling patterns between subsamples at lower body size thresholds (Tables 4 and 5), although this may be due to the small sample size in the large body size class at the 100 kg threshold (N = 36). In particular, the limb proportions of the humerus scaled differently in animals smaller than 20 kg compared to those larger than 20 kg, a pattern also noted at the 50 kg threshold. A significant difference in the proportional scaling of the femur is also noted at 50 kg. Significant differences were noted in the scaling of humeral length to body mass between individuals at the 20 kg and 50 kg threshold. As in the mammalian and reptilian comparisons, no significant differences were noted in the scaling of combined circumference and body mass between different size classes (Figure 3; Table 5).

### Independent contrast results

Overall, phylogenetically corrected scaling relationships reveal lower coefficients of determination than the raw data. The mean R^2 ^(0.9126 ± 0.0105) for the corrected data is significantly lower than that obtained from the raw data (two tailed t-test: t = -4.4721; *P *< < 0.0001). As a result, fewer significant differences were noted between mammalian clades and between mammals and reptiles [See Additional file 3, Tables S1 and S2]. Of the 80 mammalian comparisons made, two showed significant differences recovered by both the 95% confidence intervals and the likelihood ratio test. The differences include a significantly lower scaling coefficient of Carnivora compared to Glires and Ungulata, in the scaling of femur length to humerus length. Confidence intervals indicate two other differences when the data is corrected in which the humeral length of reptiles scales significantly higher than that of mammals when compared to body mass and the humeral circumference in ungulates scales higher than that of carnivorans when compared to body mass.

Most importantly, however, based on the confidence intervals, comparisons between scaling coefficients obtained from the raw data (Table 1) and the phylogenetically corrected data [See Additional file 3, Table S2] reveal only a single significant difference for the scaling of humeral proportions in Glires. Other than that comparison, the lack of significant differences between the raw data and phylogenetically corrected data suggest that phylogeny does not play a significant role in dictating the scaling patterns tested here with regards to the major weight-bearing bones in terrestrial tetrapods. For this reason, and for ease of comparison with previous limb scaling studies, further discussion will be based on results obtained from the raw data.

## Discussion

Skeletal limb morphology in vertebrates is considered to reflect a trade-off between the energetic requirements imposed by movement and the functional requirements imposed by loadings on the bone from behavioral qualities and/or body size [78,83-88]. Biomechanical studies using *in vivo *strain gauges and force platforms in mammals and birds have concluded that peak functional strains (that is, safety factors, strain at which yield or failure occur/peak functional strain) placed on a limb bone during locomotion are consistent among taxa of different size and different lifestyles (for example, terrestrial, aquatic, and aerial; [80]). However, in non-avian reptiles, safety factors are higher compared to mammals suggesting that functional strains are lower in the former [79,89,90]. Nevertheless, in order to mitigate decreases in safety factors associated with increases in body size, the architecture of the skeletal limb, such as limb robustness, cortical thickness, and/or curvature, are expected to vary [80,86,88,91].

Interspecific limb scaling patterns are often used to test theoretical biomechanical models, such as geometric, elastic, and static similarity, which predict scaling patterns based on biomechanical observations and/or assumptions [70,77,78,84,85,92-94]. These theoretical models were formally presented by McMahon [95,96], who provided empirical support for elastic scaling in terrestrial vertebrates (using ungulates as a proxy), as opposed to a strict geometric (isometric) scaling. These models were subsequently revisited by other authors who present empirical evidence that elastic similarity is restricted to ungulates with other mammals following either a geometric trend [77] or not clearly conforming to either the elastic or geometric theoretical models [85,93,94]. In general, empirical scaling studies of terrestrial mammals have found minor support for elastic similarity (see [87], for a full review). In reptiles, however, Blob [84] recovered significant support for elastic similarity in several regressions comparing limb diameters to body mass in varanids and iguanians.

The results obtained here suggest that limb scaling in mammalian and reptilian clades exhibits a great deal of variation with respect to elastic and geometric similarity, and as suggested by Christiansen [85,93], depending on the variables being compared, clades and subgroups appear to follow a variety of scaling models, and no theoretical scaling model can be used to describe all terrestrial vertebrates. However, this study suggests that elastic similarity is more prevalent than previously suggested, especially in the scaling of humeral circumference with body mass. Of the eight clades examined (Table 1), only a single group, Marsupialia, did not follow a significant allometric trend (that is, significantly different than geometric similarity), and six of the clades follow the model predicted by elastic or static similarity. In contrast, the scaling of humeral length to body mass is more closely associated with geometric similarity, as no clade follows elastic similarity, two clades follow geometric similarity, and four are negatively allometric (and therefore are below any theoretical model). Only two groups (Reptilia and Ungulata) are significantly above geometric similarity and therefore exhibit an allometric pattern whereby the length of the humerus gets shorter as body size increases, approaching a more elastic pattern. A similar pattern is present in the scaling of femoral measurements with body mass. These patterns suggest that circumference measurements tend towards allometric models suggested by McMahon [95,96], whereas length measurements follow a pattern that, in general, cannot be differentiated from isometry when compared to body mass.

The results presented here reveal that general scaling patterns of limb circumference in numerous different terrestrial vertebrates, though not always strictly elastic (as defined by McMahon), follow consistent allometric trajectories. Such allometric relationships indicate that, interspecifically, as animals get larger their limbs increase in robusticity at a higher rate compared to body mass. These changes in the architecture of the limb in relation to size support the dynamic similarity hypothesis proposed by Rubin and Lanyon [80], which predicts changes in limb structure in order to maintain safety factors [86]. The morphological changes in limb skeletal structure, as suggested by Rubin and Lanyon [80], are not the only shifts to occur with size, and likely work in concert with other shifts, such as postural and behavioral [80,84,86,88], to mitigate the response of safety factors to changes in body size. It is important to note in this respect that this study only examines the external dimensions of the bones, and that factors such as posture may influence aspects of cross-sectional bone shape (such as the relative proportions between anteroposterior and mediolateral diameters) and internal bone distribution that are not captured here. Nevertheless, the highly conserved relationships between individual and total humeral and femoral circumference and body mass suggest that in terrestrial quadrupeds external circumference measurements of the stylopodia are largely independent of posture and gait, and are most strongly associated with size, allowing us to forward the hypothesis that stylopodial circumference is more closely associated with the body mass than with the type of force (that is, compression or torsion) acting on the limb. Our results therefore present regressions that are most suitable for body mass estimation of extinct terrestrial quadrupedal vertebrates, regardless of the group under consideration.

### Stylopodial scaling as a predictor of body mass

As body mass is correlated with numerous physiological and ecological properties, (for example, [4,97]), consistent and accurate estimation of body mass in extinct taxa is important when attempting to reconstruct the dynamics of paleoecosystems and the life history of extinct taxa. The use of skeletal scaling to estimate body mass is common in extinct mammals and birds (for example, [17,41,42,45,98]); however, it is less common in extinct non-avian archosaurs and non-mammalian synapsids ([48,73,99] being notable exceptions). Scaling methods are often criticized when models are extended to more distantly related stem taxa, based on arguments such as uneven taxon sampling (ungulate bias), its applicability to animals of different gaits and limb postures, as well as its susceptibility to residual and extreme outliers [51,70,72,82]. Our dataset allows us to address these major criticisms with empirical data.

#### Ungulate uniqueness and bias

Ungulates, and specifically artiodactyls or bovids, are considered to exhibit scaling patterns distinct from those seen in other mammals. In particular, their limbs are considered to follow an elastic trend [70,77,78,93,96,100]. In addition to finding elastic trends in other mammalian clades and in reptiles, we reject previous interpretations that limb scaling in ungulates is strictly elastic. In the sample of 41 ungulates examined here (including 34 artiodactyls of which 20 are bovids), elastic similarity was recovered only in humeral circumference compared to body mass, a pattern also noted in most other clades (Table 1). Scaling of other limb measurements in ungulates either cannot be differentiated from geometric similarity, or follows allometric patterns significantly different from either theoretical model (Table 1 Sim = 0). These patterns are robust even when assessed at more exclusive levels (artiodactyls or bovids; Additional file 4, Table S3). As a result, a strict relationship between stylopodial scaling patterns and a cursorial lifestyle does not characterize ungulates to the exclusion of other mammalian clades. As such, cursorial adaptations in the limbs of ungulates may be limited to other stylopodial measurements (for example, diameter) or more distal limb bones [83,93].

**Figure 4:**
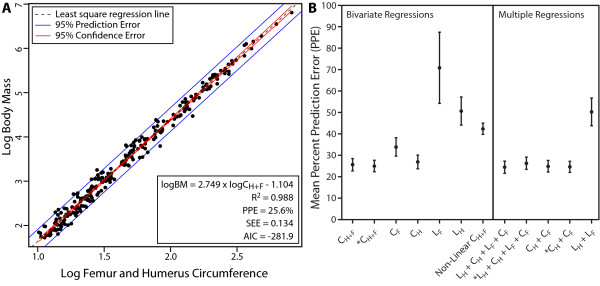
**Raw OLS regression for body mass estimation and percent prediction error of body mass proxies**. (**A**) The least-squares regression of the raw data between the log total stylopodial circumference and log body mass in a sample of 245 (talpids removed) mammals and non-avian reptiles. Regression equation shown in the format *y = mx + b*, and is presented along with its coefficient of determination (R^2^), mean percent prediction error (PPE), standard error of the estimate (SEE), and Akaike Information Criterion (AIC). (**B) **Comparison of the predictive power of several body mass proxies based on their mean PPE. The mean PPE of each proxy is represented by the black circle along with their 95% confidence error bars. The plot is divided into two sections representing the results from the bivariate and multiple regression analyses. Variables regressed against body mass are labelled along the x-axis. Labels marked with an * represent the analyses in which the data was phylogenetically adjusted through the use of a phylogenetic generalized least squares bivariate or multiple regression. C_F_, femoral circumference; C_H_, humeral circumference; L_F_, femoral length; L_H_, humeral length; OLS, ordinary least squares.

The different patterns of limb scaling observed in ungulates compared to mammals [70,77,78] are often used to cast doubt on the utility of the Anderson method to estimate body mass in extinct taxa. New data confirms some differences in limb scaling between ungulates and other mammalian clades, but only in comparisons of limb proportions (length to circumference) and length to body mass (Figure 1; Table 2). Circumference to body mass relationships reveal very high coefficients of determination and recover no significant differences between ungulates and other groups of mammals. The combined circumference of the stylopodia revealed the strongest relationship to body mass (Figure 4A) and shows that a bias towards ungulates does not significantly alter the relationship; ungulates follow the same scaling relationships of this variable to body mass as other mammals, as well as non-avian reptiles.

#### Limb scaling patterns at different gaits and limb postures

Extant terrestrial vertebrates have a variety of gaits and limb postures [79,80]. *In vivo *strain studies have also shown that in mammals, limbs of taxa of smaller body size are primarily loaded in tension, whereas compression predominates in larger taxa, resulting from postural differences with size (also related to the dynamic similarity hypothesis). Such differences are also noted in reptiles compared to mammals, in which the former hold their limbs in a sprawling fashion and hence their stylopodia are generally loaded under tension [79]. Given these postural differences, it was hypothesized that the scaling pattern of limb robusticity with body mass should vary in response to differences in limb loading [84,85]. Comparisons made here between differently sized mammals, as well as between mammals and reptiles, reveal significant differences in limb proportions, as well as in the relationships between length and body mass (Figures 2 and 3; Tables 2 and 5), and support previous studies [78,85,94]. Surprisingly, however, the relationships between limb circumference and body mass are conserved between these different groups, and no significant differences in circumferential scaling between differently sized animals and between mammals and reptiles were observed. Furthermore, we find limited evidence for geometric similarity of limb robusticity in both small and large size class samples. Instead, circumference measurements follow a generally negative allometric pattern indicating a consistent increase in circumference relative to body size in both small and large mammals. The total stylopodial circumference (Figure 4A) provides the strongest relationship (R^2 ^= 0.9861) and suggests that this variable is a strong predictor of body size for both parasagittal and sprawling taxa alike, and that combined limb circumference is not strongly correlated with limb posture and gait. These results concur with other studies on non-avian reptiles [84] and birds [101] that have shown remarkable morphological similarities of limb circumference (or diameter) between taxa with highly variable limb posture.

#### Outliers

The final criticism made towards the use of skeletal scaling methods, such as the Anderson method, to estimate body mass is related to the effect outliers have on the final predictive equation, especially at large body size where the sample size is low [82]. In the relationship between combined humeral and femoral circumference and body mass, a residual outlier test reveals that none of the largest animals in our greatly expanded dataset are residual outliers, including the buffalo, hippopotamus, and elephant (Figure 4A). The only outliers identified here appear to be related to unique ecologies, such as suspension locomotion (*Choloepus didactylus*) and burrowing (*Priodontes maximus*, *Condylura cristata*, *Parascalops breweri*), which can generally be inferred from skeletal anatomy as a potential confounding factor to mass estimation based on their highly derived limb morphologies [102]. Both representatives of Soricomorpha, *C. cristata *and *P. breweri*, are the farthest residual outliers, and, due to their especially apomorphic anatomy, will be removed from the body mass equation. Only one residual outlier, the turtle *Trachemys scripta *is difficult to explain, but its relatively high weight may be a factor of captivity or measurement error when the live weight was taken.

A recent study by Packard *et al. *[82] suggested that because of its amphibious lifestyle, *Hippopotamus amphibius *may have a high body mass compared to its limb circumference measurement. As a result, it may represent a residual outlier, which justifies the removal of *H. amphibius *from the analysis. This assertion is based on the observation that if the raw data (non-log) of Anderson *et al. *[73] is regressed using non-linear least-squares regression methods, the hippopotamus, the bison, and the elephant are all outliers. The statistical merits and flaws of logarithmically transforming data have been heavily debated (for example, [81,82,103,104]) and will not be discussed further here. However, based on the suggestions of Packard *et al. *[82], we regressed our non-log transformed expanded dataset using a non-linear least squares regression, implemented with the 'nls' function in R, and tested for potential outliers in the residual variance. The results indicate that 40 species are outliers in the non-log residual data. In order to test for potential significant effects, we removed the 40 outliers and re-ran the log-log ordinary least squares (OLS) regression, which resulted in a slope of 2.802 ± 0.055 and is statistically indistinguishable from that obtained when using the complete dataset. This suggests that these data points do not significantly affect the final result. More importantly, examination of the mean percent prediction error (PPE) indicates that despite the need for back-transformation, the log-transformed linear regression is a significantly better model for predicting body mass than a non-linear model (log PPE = 25% ± 3%; non-log PPE = 43% ± 3%; Figure 4B; two-tailed t-test: t = -8.3245, *P *< < 0.0001).

Extreme outliers, those at the upper and lower extremes of the dataset, also have the potential to significantly affect regression results. In the current dataset, there are no extreme outliers when the data is log transformed. However, as is generally the case with extant size data, there are several positive extreme outliers in the non-log dataset. Thirty-three extreme outliers are observed in the body mass and combined humeral and femoral circumference data. When these taxa are removed and the log-log analysis is re-run (*m *= 2.745 ± 0.057, *b *= -1.099 ± 0.09), the regression is virtually identical to that obtained with the total dataset. The observation that extreme positive values do not affect the log-log OLS regression is further supported by the non-significant variation in scaling coefficients between different mammalian size classes (Figure 3).

The empirical data presented here falsifies the main criticisms forwarded against skeletal-body mass regression models for predicting body mass in extinct taxa, and given the highly conserved nature of the relationship between stylopodial circumference and body mass in extant terrestrial mammals and reptiles, suggests that circumference measurements represent robust proxies of body mass that can be applied to extinct, phylogenetically and morphologically disparate quadrupedal terrestrial amniotes. The examination of eight terrestrial lissamphibian species (one caudatan and seven anurans [Additional file 1 Dataset]; not included in the final analysis) reveals that, based on their total stylopodial circumference and body mass, they plot within the range of variation present in the mammalian and reptilian dataset (Figure 2). Although at this time their small sample and range preclude any meaningful statistical comparisons between the limb scaling patterns of lissamphibians and other tetrapods, these preliminary results suggest that the conserved relationship between body mass and proximal limb bone circumference could be extended to encompass the majority of quadrupedal terrestrial tetrapods.

### Implications for body mass estimation

In extinct taxa, skeletal measurement proxies of body size are often preferred to actual body mass estimates. Of the limb measurements taken here, results suggest that the regression between the total circumference of the humerus and femur to body mass exhibits the strongest relationship, with the highest R^2 ^values, and the lowest PPE, standard error of the estimate (SEE), and Akaike Information Criterion (AIC) values of all bivariate regression models (Figure 4B; Additional file 5, Table S4). Among commonly cited proxies of size is femur length (for example, [15]). However, our analyses indicate that length measurements are generally poor indicators of size, especially compared to circumference (Figure 4B). Femur length exhibits an especially high amount of error, with a 70% mean PPE in living mammals and reptiles, compared to the 25% for the combined humeral and femoral circumference. Caution should therefore be taken when using limb length as size proxies, especially when examining taxa that encompass a wide phylogenetic bracket.

Based on our results, we propose the following scaling equation as a robust predictor of body mass in quadrupedal tetrapods:

(1) $$\text{log}\text{BM = 2}\text{.749}\cdot \text{log}{\text{C}}_{\text{H}+\text{F}}-1.104$$

where C_H+F _is the sum of humeral and femoral circumferences needed to estimate body mass. This regression exhibits a very high coefficient of determination (R^2 ^= 0.988), and a mean PPE of 25.6%. When adjusted for phylogenetic correlation/covariance between observations (that is, species) using a phylogenetic generalized least squares model, the equation is:

(2) $$\text{log}\text{BM = 2}\text{.754}\cdot \text{log}{\text{C}}_{\text{H}+\text{F}}-1.097$$

which has an almost identical mean PPE (25%) as equation 1 (Figure 4B).

In addition to examining bivariate estimates of body mass, we tested the predictive power of a variety of estimations based on multiple regressions by comparing their PPE, SEE, and AIC with those obtained from the bivariate regression of total circumference with body mass. Analyses including all proximal limb bone measurements also reveal low statistical values for both the raw data:

(3) $$\text{log}\text{BM = 0}\text{.375}\cdot \text{log}{\text{L}}_{\text{H}}+1.544\cdot \text{log}{\text{C}}_{\text{H}}-0.136\cdot \text{log}{\text{L}}_{\text{F}}+0.954\cdot \text{log}{\text{C}}_{\text{F}}-0.351$$

and the phylogenetically corrected data:

(4) $$\text{log}\text{BM = 0}\text{.212}\cdot \text{log}{\text{L}}_{\text{H}}+1.347\cdot \text{log}{\text{C}}_{\text{H}}-0.533\cdot \text{log}{\text{L}}_{\text{F}}+0.749\cdot \text{log}{\text{C}}_{\text{F}}-0.76$$

Equally low regression statistics were obtained for the multiple regression including only the circumference measurements, raw data:

(5) $$\text{log}\text{BM = 1}\text{.78}\cdot \text{log}{\text{C}}_{\text{H}}+0.939\cdot \text{log}{\text{C}}_{\text{F}}-0.215$$

phylogenetically corrected data:

(6) $$\text{log}\text{BM = 1}\text{.54}\cdot \text{log}{\text{C}}_{\text{H}}+1.195\cdot \text{log}{\text{C}}_{\text{F}}-0.234$$

None of the equations presented above are significantly better at predicting body mass than the combined humeral and femoral circumference (Equations 1 and 2); therefore, any of these equations are likely to provide robust estimates of body mass (Figure 4B). However, given that equations 2, 4, and 6 account for phylogenetic non-independence, they are likely to represent the statistical error in the data better than the non-phylogenetically corrected data.

Not surprisingly, the masses estimated for several commonly cited non-avian dinosaurs provided by Equation 2 are more consistent with estimates generated from Anderson *et al. *[73] than volumetric model-based estimates for the same taxa (Table 6). This technique is also important in that it is specimen-based, and therefore explicit and repeatable, and allows uncertainty to be expressed in the estimate. These predicted masses and prediction error ranges, when compared to previous estimates based on volumetric reconstructions [49,51,71], show that many reconstructed models underestimate body mass, sometimes significantly below that predicted by the mean PPE (Table 6). Given that life-reconstructions of extinct taxa are important for addressing several biological questions, including locomotion and weight distribution, our results provide the first objective framework with which to constrain these models and test whether their assumptions conform to the patterns seen in extant terrestrial tetrapods.

**Table 6:** Body mass estimates of some commonly cited non-avian quadrupedal dinosaurs.

Taxon	Sp #	C1962	A1985§	P1997	H1999	S2001	This study
*Iguanodon bernisartensis*	IRSNB R51	4510	7204	3200	3790	3776	8680 6510-10850
*Corythosaurus* *casuarius*	ROM 845	3820	3030	2800	-	3079	3620 2720-4530
*Protoceratops* *andrewsi*	MPC-D 100/504	177	68	164	-	23.7	79 59 - 98
*Styracosaurus* *albertensis*	AMNH 5372	3690	3649	1800	-	-	4370 3280-5460
*Triceratops horridus*	NSM PV 20379	8480	5310	6400	3938	4964	7400 5550-9250
*Stegosaurus mjosi*	SMA 0018‡	1780	4131	2200	2530	2611	4950 3720-6190
*Diplodocus longus*	USNM 10865*	10560	9061	11400	13421	19655	10940 8200-13670
*Brachiosaurus brancai*	HMN SII†	78260	29336	31500	25789∫	28655	35780 26840-44730

Body masses estimated in this study are based on the phylogenetically corrected total stylopodial circumference equation (Equation 2) and the error range is based on the 25% mean prediction error obtained from the equation. References: A1985, Anderson *et al. *[73]; C1962, Colbert [46]; H1999, Henderson [51]; P1997, Paul [71]; S2001, Seebacher [49]. Museum abbreviations in dataset file [See Additional File 1 Dataset]. * - limb measurements based off of a cast mounted at the Senckenberg Museum, Frankfurt, Germany; † - measurements taken from Anderson *et al. *[73]; ‡ - measurements from Redelstorff and Sander [145]; § - all estimates presented under A1985 are based on the equations presented in that study, but based on the limb measurements presented in dataset S1, the only exceptions are *B. brancai*, which is based on data from A1985; ∫ - estimate from Henderson [63].

## Conclusions

Body size is an important biological descriptor, and as a result, is critical to understanding the paleobiology of extinct organisms and ecosystems. This study presents an extensive dataset of extant quadrupedal terrestrial amniotes, which allows testing of the main criticisms that have been put forth against the use of scaling relationships to estimate body mass in extinct taxa. Our results demonstrate a highly conserved relationship between body mass and stylopodial circumference with minimal variation between clades and groups of different gait and size, compared over a large phylogenetic scope. This general relationship allows the estimation of body mass in extinct quadrupedal groups, and is particularly important for a wide range of paleobiological studies, including growth rates [31], metabolism[36], and energetics [105], as well as for quantifying body size changes across major evolutionary transitions that are accompanied by major changes in gait, including shifts in the early evolutionary history of archosaurs [106], and in the evolution of mammals from reptile-like basal synapsids [107,108].

## Methods

### Database construction

In order to test the hypotheses outlined in the introduction, we amassed an extensive dataset of limb bone measurements of 200 mammal and 47 non-avian reptile species from individuals that were weighed on a scale either prior to death or skeletonization; no extant body masses were estimated. For the most part, the dataset was built with newly measured specimens; however, it was augmented with published measurements from Christiansen and Harris [109] and Anderson *et al. *[73] [See Additional file 1, Dataset]. Measurements were taken from stylopodial elements, including maximum lengths and minimum circumference. Length measurements less than 150 mm were taken with digital callipers, longer dial callipers were used for measurements between 150 to 300 mm, and fiberglass measuring tape for those greater than 300 mm. Following the Anderson method, we use minimum circumference (thinnest region along the diaphysis) as a proxy for limb robusticity. In addition to reproducing the analysis presented by Anderson *et al. *[73], minimum circumference should provide a proxy of the minimum cross-sectional area of the bone and therefore be related to the overall compressive strength of the limb. Cross-sectional area was not used due to the cost of collecting this data. Moreover, circumference can be more easily measured on both extant and fossil samples, providing a larger extant dataset and a more inclusive framework for future predictive studies. Circumference measurements were taken with thin paper measuring tapes of different widths, depending on the size of the specimen being measured. All measurements were taken from both sides of the specimen, where possible, and averaged. Specimens measured are of adult body size. For most of the mammalian sample, the ontogenetic status of the specimen was determined based on the level of epiphyseal fusion. For the non-avian reptile sample, as well as some of the largest mammals, maturity was established by verifying that the body mass of the measured specimen is similar to published reports of average body masses for that species (for example, [84,110-112]). In general, only a single specimen of each species could be obtained; however, in instances where more than one adult individual was available, the largest individual was used in this study. In these cases, none of the exemplars used seem unusually large compared to the reported adult body mass in that species. Finally, this study compares taxa with different growth strategies (mammals have determinate growth whereas growth in reptiles is generally considered indeterminate, but asymptotic [113]) that may result in differences in size structuring within and between populations of taxa with these different strategies. If, and/or how, these differences affect limb to body mass scaling analyses is unknown at this time. However, the masses of the reptiles used here fall within the range of what is considered typical for an adult of each species, and, given our large sample and the nature of our results (see below), we expect that these effects will be minimal, yet may warrant future consideration.

#### Taxon sampling

Taxa were chosen based on three criteria: 1) The dataset must include a large range in body mass, so that size-related postural differences can be assessed [83,114]. We significantly expand upon the dataset of Anderson *et al. *[73], especially for large bodied mammalians species, to better represent the range of variation in limb proportions at large sizes and address the contention that certain large taxa are residual outliers [82]. Due to the limitations of measuring limb bone circumference, taxa below 50 g were not included in this study. 2) The sample must encompass a wide phylogenetic scope, so that most major mammalian and reptilian clades are sampled. 3) The sample must include taxa from a broad spectrum of lifestyles. Our study focuses on terrestrial taxa; however, we have also included mammalian or reptilian taxa with specialized lifestyles that have the potential to affect limb proportions and their relationship with body size. These include saltators (Macropodidae), brachiators (*Hylobates lar*, and *Pongo pygmaeus*), burrowers (for example, Talpidae), and amphibious taxa (Hippopotamidae and Crocodylia). The former three categories are associated with salient morphological features that allow these lifestyles to be recognized in the fossil record; however, the amphibious nature of several extinct taxa remains uncertain, and may affect how limb measurements scale with body mass due to the effects of buoyancy.

Avian taxa were not included in the current study because they are bipedal. The forces exerted by body mass in a biped are transmitted through two limbs compared to four in a quadruped, and therefore direct comparisons of limb to body mass scaling between birds and quadrupedal tetrapods are difficult to interpret. A small sample of lissamphibians (one caudatan and seven anurans) for which live body mass is known was examined in this study. Unfortunately, the current sample size does not provide enough power to make meaningful slope and intercept comparisons, and lissamphibians are not included in the main comparisons presented in the results section.

### Statistical analyses

The distribution of the variables used in this study are all positively skewed and, therefore, highly different from a normal distribution; as such all variables were logarithmically transformed (at base 10) to approximate a log-normal distribution. In addition to normality, log transforming reduces the level of heteroscedasticity in the data set, minimizes the effect of extreme outliers, and allows for the visualization of data in a linear fashion, which simplifies the visual comparisons of slopes [81,115]. The benefits and complications regarding the application of log transformation in predictive scaling relationships were recently debated by Packard *et al. *[82] and Cawley and Janacek [104]. We agree with the latter study, which demonstrated that log-transformed data is preferred for this type of analysis as it assigns an equal weight to all data points in a regression, rather than upper extreme values and, furthermore, residuals are not significantly related to size [104].

#### Interspecific limb scaling

All measurements were incorporated into a variety of bivariate plots and analyzed using the SMA line-fitting method (also known as Reduced Major Axis) [116]. The analyses compare a variety of measurements, including: 1) limb proportions, such as femur length to humerus length and humerus/femur length to circumference; and 2) limb measurements to body mass, such as humerus/femur length versus body mass and humerus/femur circumference versus body mass. All SMA analyses were conducted using the open-source software R [117] and the package 'smatr' [116,118].

To address the criticisms raised against the Anderson method, subgroups within the data were compared. These include comparisons between mammalian clades for which a sample size greater than ten could be obtained, such as Ungulata, Carnivora, Marsupialia, Euarchonta, and Glires. In addition, comparisons were made between different size classes. Size class comparisons were based on three body mass thresholds: 20 kg, which was previously used by Economos [94] to show differential scaling in mammals, and it is also thought to represent the lower size limit for migratory mammals and hence may affect limb scaling patterns [4]; 50 kg, a threshold at which mammalian limb scaling has been previously noted to vary [93]; and 100 kg, previously used by Bertram and Biewener [78], and which allows better representation of the large-bodied portion of the dataset.

Fitted lines of different subsamples were compared based on the 95% confidence intervals of the slope and intercept, and differences were considered to be significant when intervals did not overlap. However, given that statistical significance can still be obtained even though confidence intervals overlap [81], we conducted a series of pairwise comparisons of the slopes and intercepts using a likelihood ratio test and a t-test, respectively. These tests have the added benefit that they can be corrected for errors associated with multiple comparisons using the FDR, an approach that, as far as we are aware, cannot be applied to confidence intervals [119,120]. The likelihood ratio test was implemented with the 'smatr' package [116,118]. Conventional methods for comparing intercepts (for example, ANCOVA, Wald statistic, and traditional t-tests) alter the original intercepts by forcing a common slope to each group being analyzed [115,116]. Although this may make statistical sense [116], it involves permuting the best fit-line away from the original biological data. As a result, here we compare intercepts using a two-tailed t-test based on equation 18.25 of Zar [115]:

$$\text{t}=\left(b_{1}-b_{2}\right)/\text{S}{\text{E}}_{\text{SMA}}$$

where *b*_1 _and *b*_2 _represent the pair of intercepts being compared, and SE_SMA _is the standard error of the difference in SMA intercepts, calculated as per equation 18.26 of Zar [115]. Comparing intercepts using this method has the added benefit of allowing comparisons of y-values along the true SMA lines at x-values other than 0. This is advisable when comparing biological scaling lines because first, the intercept at x = 0 is an extrapolation of the line beyond the range of the data [115], but perhaps more importantly given the type of data used here, a value of x = 0 is biologically meaningless. As a result, in addition to presenting the results of the t-test at the true intercept, we compare y-values at the minimum value of the total dataset along the x-axis using the same t-test method. The results of the two intercept comparison methods described above are presented, and all *P*-values are corrected using the FDR [119,120], implemented with the 'p.adjust' function in R. In total, 14 pairwise comparisons are made for each analysis.

In addition to comparing limb scaling patterns between different groups, scaling coefficients were used to test theoretical scaling models, such as geometric (GS), elastic (ES), and static (SS) similarity [95,96]. The models predict that under GS: circumference ∝ length; mass ∝ length^3^; mass ∝ circumference^3^, under ES: circumference ∝ length^1.5^; mass ∝ length^4^; mass ∝ circumference^2/3^, and finally under SS: circumference ∝ length^2^; mass ∝ length^5^; mass ∝ circumference^2.5^. These models were tested against the empirical slopes obtained in this study using the method described by Warton et al. [116].

#### Phylogenetic independent contrasts

In addition to plotting the raw data, as was done by Anderson *et al. *[73], we calculated the phylogenetic independent contrasts (PIC) for the entire dataset in order to correct for non-independence of the raw data as a result of common ancestry [121]. We compared the scaling coefficients from the raw and phylogenetically corrected data to test if non-independence significantly alters the scaling patterns obtained from the raw data. The phylogenetic tree [See Additional file 6, Figure S1] was constructed in Mesquite [122], based on recent phylogenetic analyses obtained for extant Mammalia [123], and non-avian reptiles [124-130]. Branch lengths are measured in millions of years. For the mammalian portion of the phylogeny we used the branch lengths of Bininda-Emonds *et al. *[123]. Branch lengths in the reptile portion of the tree were largely calculated using molecular estimates of divergence times [131-138]. However, species-level divergence times of some taxa, such as turtles, are poorly constrained, and as a result, we estimated the branch lengths based on the oldest known fossil occurrence for the species or genus obtained from the Paleobiology Database http://paleodb.org/.

Both theoretical and empirical studies of PIC state that in order for contrasts to receive equal weighting and thereby conform to the assumptions stipulated by parametric analyses and statistics, branch lengths must be adjusted so that contrasts are standardized, and therefore have a non-significant relationship with their standard deviation [139]. The criterion was not met by the raw branch lengths, but was obtained by transforming the branch lengths by their natural log. Branch lengths were assigned and transformed in Mesquite and the tree file was imported into R, where contrasts were calculated using the 'APE' package [140]. A best fit line was calculated for the contrasts using a SMA in the package 'smatr' [116], which allows for the line to pass through the origin, as stipulated by Garland *et al. *[139]. The PIC slopes for the entire dataset and subsets (as described above) were compared to slopes obtained from the raw data using the 95% confidence intervals.

#### Body mass estimation

In order to provide the best estimation parameter for body mass, a Model I (OLS) regression analysis is preferred. It is the most appropriate model for estimating a value of y based on x, as it accounts for the complete error of the y variable that can be explained by the x variable [81,141]. The analysis was performed on the entire dataset (N = 247) between body mass and a variety of limb measurements in order to test for the best predictor. The 'goodness of fit' of a predictor was examined based on the commonly used coefficient of determination (R^2^); however, this value is considered a poor representation of the strength of a regression, due largely to its strong association with sample size [103]. Therefore, given the large dataset presented here, we provide three additional metrics, including the SEE, the PPE, and the AIC. The mean PPE is perhaps the best metric of regression strength for these types of analyses as it deals with the predictive strength of the relationship in relation to the non-logged data. In addition, the PPE has the added benefit of allowing for calculation of confidence intervals around the mean PPE, and therefore facilitates comparison between the mean PPE of different models.

In addition to the OLS bivariate regression outlined above, we included all limb measurements into a suite of multiple regression analyses and, given that this technique is highly recommended [43,47,142], tested if they are significantly better predictors of body mass than bivariate regressions. The predictive accuracy of each analysis was compared using SEE, PPE, and AIC. Finally, because none of the bivariate or multiple regressions account for correlation and covariance of morphology between taxa as a result of phylogenetic history, we re-analyzed the data using a phylogenetic generalized least squares approach [143], a method recently applied to estimate body mass in extinct bovids [144]. Application of this method is based on the same phylogenetic tree, branch lengths [See Additional file 6, Figure S1], and a Brownian motion model of evolution. This approach was implemented using the 'APE' and 'nlme' packages in R.

## Abbreviations

AIC: Akaike Information Criterion; ES: elastic similarity; FDR: false discovery rate; GS: geometric similarity; OLS: ordinary least squares; PIC: phylogenetic independent contrasts; PPE: percent prediction error; SEE: standard error of the estimate; SMA: standardized major axis; SS: static similarity.

## Competing interests

The authors declare that they have no competing interests.

## Authors' contributions

NEC and DCE conceived and designed the study, collected the data, and drafted the manuscript. NEC carried out all the data analyses.

## Supplementary Material

Additional file 1 **Limb measurement and body mass data**. Table of measurements of all the extant taxa used in the present study, as well as the limb measurements of the non-avian dinosaurian taxa shown in Table 6. Click here for file

Additional file 2 **Table S1**. Raw and PIC stylopodial scaling in a subset of the mammalian dataset and non-avian reptiles. Mammalian subset corresponds to all taxa < 168 kg in order to better approximate body mass range in the sample of non-avian reptiles. Standardized Major Axis equation shown in the format ***y = mx + b ***(***b ***= 0 in PIC). The particular theoretical scaling model (Sim.) followed by the slope is represented by G, geometric similarity, E, elastic similarity, or S, static similarity. Scaling patterns that fall between models are represented by > or <, and those that do not follow any pattern (that is, above or below all predicted models) are represented by a 0. Click here for file

Additional file 3 **Table S2**. Phylogenetically corrected stylopodial scaling in mammals and non-avian reptiles. Scaling equation shown in the format *y = mx*. The particular theoretical scaling model (Sim.) followed by the slope is represented by G, geometric similarity, E, elastic similarity, or S, static similarity. Scaling patterns that fall between models are represented by > or <, and those that do not follow any pattern (that is, above or below all predicted models) are represented by a 0. Click here for file

Additional file 4 **Table S3**. Raw and PIC stylopodial scaling in Artiodactyla and Bovidae. Standardized Major Axis equation shown in the format *y = mx + b *(*b *= 0 in PIC). The particular theoretical scaling model (Sim.) followed by the slope is represented by G, geometric similarity, E, elastic similarity, or S, static similarity. Scaling patterns that fall between models are represented by > or <, and those that do not follow any pattern (that is, above or below all predicted models) are represented by a 0. Click here for file

Additional file 5 **Table S4**. Predictive power of various body mass estimation equations. Bivariate and multiple regression statistics for various body mass proxies discussed here (that is, circumference and length of the humerus and femur). Statistics include the Percent Prediction Error (PPE), along with its upper and lower 95% PPE Confidence Intervals (PPE CI), the Standard Error of the Estimate (SEE), the Coefficient of Determination (R^2^), and the Akaike Information Criterion Score (AIC). Click here for file

Additional file 6 **Figure S1**. Phylogenetic tree of mammalian and reptilian taxa included in this study. Topology is based on multiple published analyses mentioned in the text. Numbers indicate the branch lengths used in this study, measured in millions of years. Terminal branch lengths are most often given next to the species name. Click here for file

## References

[B1] Hemmingsen AM Energy metabolism as related to body size and respiratory surfaces, and its evolution Steno Memorial Hospital and Nordinsk Insulin Laboratosium 1960 9 6 110

[B2] Kleiber M Body size and metabolic rate Physiol Rev 1947 27 4 511 541 20267758 10.1152/physrev.1947.27.4.511

[B3] Gillooly JF Brown JH West GB Savage VM Charnov EL Effects of size and temperature on metabolic rate Science 2001 293 2248 2251 10.1126/science.1061967 11567137

[B4] Peters RH The Ecological Implications of Body Size 1983 New York: Cambridge University Press

[B5] Gillooly JF Charnov EL West GB Savage VM Brown JH Effects of size and temperature on developmental time Nature 2002 417 70 73 10.1038/417070a 11986667

[B6] Brown JH Marquet PA Taper ML Evolution of body size: consequences of an energetic definition of fitness Am Nat 1993 142 573 584 10.1086/285558 19425961

[B7] McClain CR Boyer AG Biodiversity and body size are linked across metazoans Proc R Soc Lond B Biol Sci 2009 276 2209 2215 10.1098/rspb.2009.0245 PMC2677615 19324730

[B8] Calder WAI Size, Function, and Life History 1984 Cambridge, MA: Harvard University Press

[B9] Damuth J Population density and body size in mammals Nature 1981 290 699 700 10.1038/290699a0

[B10] Burness GP Diamond J Flannery T Dinosaurs, dragons, and dwarfs: the evolution of maximal body size Proc Natl Acad Science USA 2001 98 14518 14523 10.1073/pnas.251548698 PMC64714 11724953

[B11] Gaston KJ Blackburn TM Range size-body size relationships: evidence of scale dependence Oikos 1996 1996 479 485

[B12] Capellini I Gosling LM Habitat primary production and the evolution of body size within the hartebeest clade Biol J Linnean Soc 2007 92 431 440 10.1111/j.1095-8312.2007.00883.x

[B13] Butler RJ Goswami A Body size evolution in Mesozoic birds: little evidence for Cope's rule J Evol Biol 2008 21 1673 1682 10.1111/j.1420-9101.2008.01594.x 18691237

[B14] Carrano MT Rogers KC, Wilson JA The evolution of sauropod locomotion: morphological diversity of a secondarily quadrupedal radiation The Sauropods: Evolution and Paleobiology 2005 Berkeley, CA: University of California Press 229 251

[B15] Carrano MT Carrano MT, Blob RW, Gaudin TJ, Wible JR Body-size evolution in the Dinosauria Amniote Paleobiology: Perspectives on the Evolution of Mammals, Birds, and Reptiles 2006 Chicago, IL: University of Chicago Press 225 268

[B16] Hone DWE Keesey TM Pisani D Purvis A Macroevolutionary trends in the Dinosauria: Cope's rule J Evol Biol 2005 18 587 595 10.1111/j.1420-9101.2004.00870.x 15842488

[B17] Hone DWE Dyke GJ Haden M Benton MJ Body size evolution in Mesozoic birds J Evol Biol 2008 21 618 624 10.1111/j.1420-9101.2007.01483.x 18194232

[B18] Therrien F Henderson DM My theropod is bigger than yours...or not: estimating body size from skull length in theropods J Vertebrate Paleontol 2007 27 108 115 10.1671/0272-4634(2007)27[108:MTIBTY]2.0.CO;2

[B19] Turner AH Pol D Clarke JA Erickson GM Norell MA A basal dromaeosaurid and size evolution preceding avian flight Science 2007 317 1378 1381 10.1126/science.1144066 17823350

[B20] Laurin M The evolution of body size, Cope's rule and the origin of amniotes Syst Biol 2004 53 594 622 10.1080/10635150490445706 15371249

[B21] Finarelli JA Flynn JJ Ancestral state reconstruction of body size in the Caniformia (Carnivora, Mammalia): the effects of incorporating data from the fossil record Syst Biol 2006 55 301 313 10.1080/10635150500541698 16611601

[B22] Finarelli JA Hierarchy and the reconstruction of evolutionary trends: evidence for constraints on the evolution of body size in terrestrial caniform carnivorans (Mammalia) Paleobiology 2008 34 553 563 10.1666/07078.1

[B23] Hopson JA Relative brain size and behavior in archosaurian reptiles Annu Rev Ecol Systematics 1977 8 429 448 10.1146/annurev.es.08.110177.002241

[B24] Hopson JA Gans C, Northcutt RG, Ulinski P Paleoneurology Biology of the Replilia, Neurology A 1979 9 New York: Academic Press 39 146

[B25] Jerison HJ Brain evolution and dinosaur brains Am Nat 1969 103 575 588 10.1086/282627

[B26] Jerison HJ Evolution of the Brain and Intelligence 1973 New York: Academic Press

[B27] Varricchio DJ Moore JR Erickson GM Norell MA Jackson FD Borkowski JJ Avian paternal care had dinosaur origin Science 2008 322 1826 1828 10.1126/science.1163245 19095938

[B28] Janis CM Carrano MT Scaling of reproductive turnover in archosaurs and mammals: why are large terrestrial mammals so rare? Ann Zool Fennici 1992 28 201 216

[B29] Varricchio DJ Jackson F Borkowski JJ Horner JR Nests and egg clutches of the dinosaur *Troodon formosus *and the evolution of avian reproductive traits Nature 1997 385 247 250 10.1038/385247a0

[B30] Erickson GM Assessing dinosaur growth patterns: a microscopic revolution Trends Ecol Evol 2005 20 677 684 10.1016/j.tree.2005.08.012 16701457

[B31] Erickson GM Rogers KC Yerby SA Dinosaurian growth patterns and rapid avian growth rates Nature 2001 412 429 433 10.1038/35086558 11473315

[B32] Christiansen P Long bone scaling and limb posture on non-avian theropods: evidence for differential allometry J Vertebrate Paleontol 1999 19 666 680 10.1080/02724634.1999.10011180

[B33] Carrano MT Locomotion in non-avian dinosaurs: integrating data from hindlimb kinematics, in vivo strains, and bone morphology Paleobiology 1998 24 450 469

[B34] Pontzer H Allen V Hutchinson JR Biomechanics of running indicates endothermy in bipedal dinosaurs PLoS One 2009 4 1 9 10.1371/journal.pone.0005361 PMC2772121 19911059

[B35] Bakker RT Anatomical and ecological evidence of endothermy in dinosaurs Nature 1972 239 81 85

[B36] Gillooly JF Allen AP Charnov EL Dinosaur fossils predict body temperatures PLoS Biology 2006 4 e248 10.1371/journal.pbio.0040248 16817695 PMC1489189

[B37] Head JJ Bloch JI Hastings AK Bourque JR Cadena EA Herrera FA Polly PD Jaramillo CA Giant boid snake from the Palaeocene neotropics reveals hotter past equatorial temperatures Nature 2009 457 715 718 10.1038/nature07671 19194448

[B38] Franz R Hummel J Kiensle E Kölle P Gunga H-C Clauss M Allometry of visceral organs in living amniotes and its implications for sauropod dinosaurs Proc R Soc Lond B Biol Sci 2009 276 1731 1736 10.1098/rspb.2008.1735 PMC2660986 19324837

[B39] Farlow JO A consideration of the trophic dynamics of a Late Cretaceous large-dinosaur community (Oldman Formation) Ecology 1976 57 841 857 10.2307/1941052

[B40] Peczkis J Implications of body-mass estimates for dinosaurs J Vertebrate Paleontol 1994 14 520 533

[B41] Damuth J, MacFadden BJ Body Size in Mammalian Paleobiology: Estimation and Biological Implications 1990 Cambridge, UK: Cambridge University Press

[B42] Millien V Bovy H When teeth and bones disagree: body mass estimates in a giant extinct rodent J Mammal 2010 91 11 18 10.1644/08-MAMM-A-347R1.1

[B43] De Esteban-Trivigno S Mendoza M De Renzi M Body mass estimation in Xenarthra: a predictive equation suitable for all quadrupedal terrestrial placentals? J Morphol 2008 269 1276 1293 10.1002/jmor.10659 18655156

[B44] Gingerich PD Prediction of body mass in mammalian species from long bone lengths and diameters Contributions from the Museum of Paleontology, University of Michigan 1990 28 79 92

[B45] Campbell KE Marcus L The relationships of hindlimb bone dimensions to body weight in birds Natural History Museum of Los Angeles County Science Series 1992 36 395 412

[B46] Colbert EH The weights of dinosaurs American Museum Novitates 1962 2076 1 16

[B47] Christiansen P Fariña RA Mass prediction in theropod dinosaurs Hist Biol 2004 16 85 92 10.1080/08912960412331284313

[B48] Hurlburt G Comparison of body mass estimation techniques, using recent reptiles and the pelycosaur *Edaphosaurus boanerges* J Vertebrate Paleontol 1999 19 338 350 10.1080/02724634.1999.10011145

[B49] Seebacher F A new method to calculate allometric length-mass relationships of dinosaurs J Vertebrate Paleontol 2001 21 51 60 10.1671/0272-4634(2001)021[0051:ANMTCA]2.0.CO;2

[B50] Gunga H-C Kirsch K Rittweger J Röcker L Clarke A Albertz J Wiedemann A Mokry S Suthau T Wehr A Body size and body volume distribution in two sauropods from the Upper Jurassic of Tendaguru (Tanzania) Mitteilungen aus dem Museum für Naturkunde der Humboldt-Universität Berlin, Geowissenschaftliche Reihe 1999 2 91 102

[B51] Henderson DM Estimating the masses and centers of mass of extinct animals by 3-D mathematical slicing Paleobiology 1999 25 88 106

[B52] Bates KT Manning PL Hodgetts D Sellers WI Estimating mass properties of dinosaurs using laser imaging and 3D computer modelling PLoS One 2009 4 e4532 10.1371/journal.pone.0004532 19225569 PMC2639725

[B53] Gunga H-C Suthau T Bellmann A Andreas F Schwanebeck T Stoinski S Trippel T Kirsch K Hellwich O Body mass estimations for *Plateosaurus engelhardti *using laser scanning and 3D reconstriction methods Naturwissenschaften 2007 94 623 630 10.1007/s00114-007-0234-2 17356876

[B54] Gunga H-C Suthau T Bellmann A Stoinski S Friedrich A Trippel T Kirsch K Hellwich O A new body mass estimation of *Brachiosaurus brancai *Janensch, 1914 mounted and exhibited at the Museum of Natural History (Berlin, Germany) Fossil Record 2008 11 33 38 10.1002/mmng.200700011

[B55] Motani R Estimating body mass from silhouettes: testing the assumption of elliptical body cross-sections Paleobiology 2001 27 735 750 10.1666/0094-8373(2001)027<0735:EBMFST>2.0.CO;2

[B56] Gunga H-C Kirsch K Rittweger J Clarke A Albertz J Wiedemann A Wehr A Heinrich W-D Schultze H-P Dimensions of *Brachiosaurus brancai*, *Dicraeosaurus hansemanni *and *Diplodocus carnegii *and their implications for gravitational physiology Adaptation Biol Med 2002 3 156 169

[B57] Grand TI Damuth J, MacFadden BJ The functional anatomy of body mass Body Size in Mammalian Paleobiology: Estimation and Biological Implications 1990 Cambridge, UK: Cambridge University Press 39 47

[B58] Benson RBJ Butler RJ Carrano MT O'Connor PM Air-filled postcranial bones in theropod dinosaurs: physiological implications and the 'reptile'-bird transition Biol Rev 2011 87 168 193 21733078 10.1111/j.1469-185X.2011.00190.x

[B59] Wedel MJ Vertebral pneumaticity, air sacs, and the physiology of sauropod dinosaurs Paleobiology 2003 29 243 255 10.1666/0094-8373(2003)029<0243:VPASAT>2.0.CO;2

[B60] Hazlehurst GA Rayner JMV Flight characteristics of Triassic and Jurassic Pterosauria: an appraisal based on wing shape Paleobiology 1992 18 447 463

[B61] Hutchinson JR Bates KT Molnar J Allen V Makovicky PJ A computational analysis of limb and body dimensions in *Tyrannosaurus rex *with implications for locomotion, ontogeny, and growth PLoS One 2011 6 e26037 10.1371/journal.pone.0026037 22022500 PMC3192160

[B62] Bates KT Falkingham PL Breithaupt BH Hodgetts D Sellers WI Manning PL How big was 'Big Al'? Quantifying the effects of soft tissue and osteological unknowns on mass predictions for *Allosaurus *(Dinosauria: Theropoda) Palaeontologia Electronica 2009 12 33

[B63] Henderson DM Tipsy punters: sauropod dinosaur pneumaticity, buoyancy and aquatic habits Biol Lett 2004 271 S180 S183 10.1098/rsbl.2003.0136 PMC1810024 15252977

[B64] Henderson DM Pterosaur body mass estimates from three-dimensional mathematical slicing J Vertebrate Paleontol 2010 30 768 785 10.1080/02724631003758334

[B65] Henderson DM Burly gaits: centers of mass, stability, and the trackways of sauropod dinosaurs J Vertebrate Paleontol 2006 26 907 921 10.1671/0272-4634(2006)26[907:BGCOMS]2.0.CO;2

[B66] Hohn B Klein N, Remes K, Gee CT, Sander PM Walking with the shoulder of giants: biomechanical conditions in the tetrapod shoulder girdle as a basis for Sauropod shoulder reconstruction Biology of the Sauropod Dinosaurs: Understanding the Life of Giants 2011 Bloomington, ID: Indiana University Press 182 196

[B67] Mallison H Klein N, Remes K, Gee CT, Sander PM Plateosaurus in 3D: how CAD models and kinetic-dynamic modeling bring an extinct animal to life Biology of the Sauropod Dinosaurs: Understanding the Life of Giants 2011 Bloomington, ID: Indiana University Press 219 236

[B68] Mallison H Klein N, Remes K, Gee CT, Sander PM Rearing giants: kinetic-dynamic modeling of sauropod bipedal and tripodal poses Biology of the Sauropod Dinosaurs: Understanding the Life of Giants 2011 Bloomington, ID: Indiana University Press 237 250

[B69] Fortelius M Kappelman J The largest land mammal ever imagined Zool J Linnean Soc 1993 108 85 101 10.1111/j.1096-3642.1993.tb02560.x

[B70] Carrano MT Implications of limb bone scaling, curvature and eccentricity in mammals and non-avian dinosaurs J Zool 2001 254 41 55 10.1017/S0952836901000541

[B71] Paul G Wolberg DL, Stump E, Rosenberg GD Dinosaur models: the good, the bad, and using them to estimate the mass of dinosaurs Dinofest International 1997 Arizona State University: The Academy of Natural Sciences 39 45

[B72] Alexander RM Dynamics of Dinosaurs and Other Extinct Giants 1989 New York: Columbia University Press

[B73] Anderson JF Hall-Martin A Russell DA Long-bone circumference and weight in mammals, birds and dinosaurs J Zool Soc Lond A 1985 207 53 61

[B74] Casinos A Bipedalism and quadrupedalism in *Megatherium*: an attempt at biomechanical reconstruction Lethaia 1996 29 87 96 10.1111/j.1502-3931.1996.tb01842.x

[B75] Hutchinson JR Ng-Thow-Hing V Anderson FC A 3D interactive method for estimating body segmental parameters in animals: application to the turning and running performance of *Tyrannosaurus rex* J Theoret Biol 2007 246 660 680 10.1016/j.jtbi.2007.01.023 17363001

[B76] Lehman TM Woodward HN Modeling growth rates for sauropod dinsoaurs Paleobiology 2008 34 264 281 10.1666/0094-8373(2008)034[0264:MGRFSD]2.0.CO;2

[B77] Alexander RM Jayes AS Maloiy GMO Wathuta EM Allometry of the limb bones of mammals from shrews (*Sorex*) to elephant (*Loxodanta*) J Zool 1979 189 305 314

[B78] Bertram JEA Biewener AA Differential scaling of the long bones in the terrestrial Carnivora and other mammals J Morphol 1990 204 157 169 10.1002/jmor.1052040205 2348461

[B79] Blob RW Biewener AA *In vivo *locomotor strain in the hindlimb bones of *Alligatos mississippiensis *and *Iguana iguana*: implications for the evolution of limb bone safety factor and non-sprawling limb posture J Exp Biol 1999 202 1023 1046 10101104 10.1242/jeb.202.9.1023

[B80] Rubin CT Lanyon LE Dynamic strain similarity in vertebrates: an alternative to allometric limb bone scaling J Theoret Biol 1984 107 321 327 10.1016/S0022-5193(84)80031-4 6717041

[B81] Sokal RR Rohlf FJ Biometry: The Principles and Practice of Statistics in Biological Science 1969 San Francisco, CA: W. H. Freeman and Company

[B82] Packard GC Boardman TJ Birchard GF Allometric equations for predicting body mass of dinosaurs J Zool 2009 279 102 111 10.1111/j.1469-7998.2009.00594.x

[B83] Carrano MT What, if anything, is a cursor? Categories versus continua for determining locomotor habit in mammals and dinosaurs J Zool 1999 247 29 42 10.1111/j.1469-7998.1999.tb00190.x

[B84] Blob RW Interspecific scaling of the hindlimb skeleton in lizards, crocodilians, felids and canids: does limb bone shape correlate with limb posture? J Zool 2000 250 507 531 10.1111/j.1469-7998.2000.tb00793.x

[B85] Christiansen P Scaling of mammalian long bones: small and large mammals compared J Zool 1999 247 333 348 10.1111/j.1469-7998.1999.tb00996.x

[B86] Rubin CT Lanyon LE Limb mechanics as a function of speed and gait: a study of functional strains in the radius and tibia of horse and dog J Evol Biol 1982 101 187 211 10.1242/jeb.101.1.187 7166694

[B87] Garcia GJM da Silva JKL Review: interspecific allometry of bone dimensions: a review of the theoretical models Phys Life Rev 2006 3 188 209 10.1016/j.plrev.2006.07.002

[B88] Clemente CJ Withers PC Thompson G Lloyd D Evolution of limb bone loading and body size in varanid lizards J Exp Biol 2011 214 3013 3020 10.1242/jeb.059345 21865513

[B89] Blob RW Biewener AA Mechanics of limb bone loading during terrestrial locomotion in the green iguana (*Iguana iguana*) and American alligator (*Alligatos mississippiensis*) J Exp Biol 2001 204 1099 1122 11222128 10.1242/jeb.204.6.1099

[B90] Butcher MT Espinoza NR Cirilo SR Blob RW In vivo strains in the femur of river cooter turtles (*Pseudemys concinna*) during terrestrial locomotion: tests of force-platform models of loading mechanics J Exp Biol 2008 211 2397 2407 10.1242/jeb.018986 18626073

[B91] Biewener AA Scaling body support in mammals: limb posture and muscle mechanics Science 1989 245 45 48 10.1126/science.2740914 2740914

[B92] Biewener AA Allometry of quadrupedal locomotion: the scaling of duty factor, bone curvature and limb orientation to body size J Exp Biol 1983 105 147 171 6619724 10.1242/jeb.105.1.147

[B93] Christiansen P Scaling of the limb long bones to body mass in terrestrial mammals J Morphol 1999 239 167 190 10.1002/(SICI)1097-4687(199902)239:2<167::AID-JMOR5>3.0.CO;2-8 9951716

[B94] Economos AC Elastic and/or geometric similarity in mammalian design? J Theoret Biol 1983 103 167 172 10.1016/0022-5193(83)90206-0 6621067

[B95] McMahon T Size and shape in biology Science 1973 179 1201 1204 10.1126/science.179.4079.1201 4689015

[B96] McMahon TA Using body size to understand the structural design of animals: quadrupedal locomotion J Appl Physiol 1975 39 619 627 1194153 10.1152/jappl.1975.39.4.619

[B97] Marquet PA Quiñones RA Abades S Labra F Tognelli M Arim M Rivadeneira M Review: scaling and power-laws in ecological systems J Exp Biol 2005 208 1749 1769 10.1242/jeb.01588 15855405

[B98] Boyer AG Jetz W Biogeography of body size in Pacific island birds Ecography 2010 33 369 379

[B99] Young MT Bell MA De Andrade MB Brussatte SL Body size estimation and evolution in metriorhynchid crocodylomorphs: implications for species diversification and niche partitioning Zool J Linnean Soc 2011 163 1199 1216 10.1111/j.1096-3642.2011.00734.x

[B100] McMahon TA Allometry and biomechanics: limb bones in adult ungulates Am Naturalist 1975 109 547 563 10.1086/283026

[B101] Farke AA Alicea J Femoral strength and posture in terrestrial birds and non-avian theropods Anat Rec 2009 292 1406 1411 10.1002/ar.20963 19711474

[B102] Bou J Casinos A Ocaña J Allometry of the limb long bones of insectivores and rodents J Morphol 1987 192 113 123 10.1002/jmor.1051920204 3599079

[B103] Smith RJ Allometric scaling in comparative biology: problems of concept and method Am J Physiol Regul Integr Comp Physiol 1984 246 R152 R160 10.1152/ajpregu.1984.246.2.R152 6696141

[B104] Cawley GC Janacek GJ On allometric equations for predicting body mass of dinosaurs J Zool 2009 280 355 361

[B105] Finnegan S Droser ML Body size, energetics, and the Ordovician restructuring of marine ecosystems Paleobiology 2008 34 342 359 10.1666/07074.1

[B106] Hutchinson JR The evolution of locomotion in archosaurs C R Palevol 2006 5 519 530 10.1016/j.crpv.2005.09.002

[B107] Blob RW Evolution of hindlimb posture in nonmammalian therapsids: biomechanical tests of paleontological hypotheses Paleobiology 2001 27 14 38 10.1666/0094-8373(2001)027<0014:EOHPIN>2.0.CO;2

[B108] Fröbisch J Locomotion in derived dicynodonts (Synapsids, Anomodontia): a functional analysis of the pelvis girdle and hind limb of *Tetragonia njalilus* Can J Earth Sci 2006 43 1297 1308 10.1139/e06-031

[B109] Christiansen P Harris JM Body size of *Smilodon *(Mammalia: Felidae) J Morphol 2005 266 369 384 10.1002/jmor.10384 16235255

[B110] Andrews RM Pough FH Metabolism of squamate reptiles: allometric and ecological relationships Physiol Zool 1985 58 214 231

[B111] Woodward AR White JH Linda SB Maximum size of the alligator (*Alligator mississippiensis*) J Herpetol 1995 29 507 513 10.2307/1564733

[B112] Jones KE Bielby J Cardillo M Fritz SA O'Dell J Orme CDL Safi K Sechrest W Boakes EH Carbone C PanTHERIA: a species-level database of life history, ecology, and geography of extant and recently extinct mammals Ecology 2009 90 2648 10.1890/08-1494.1

[B113] Andrews RM Gans C, Pough FH Patterns of growth in reptiles Biology of the Reptilia: Physiology D 1982 13 London, UK: Academic Press 273 320

[B114] Jenkins FA Limb posture and locomotion in the Virginia opossum (*Didelphis marsupialis*) and in other non-cursorial mammals J Zool 1971 165 303 315

[B115] Zar JH Calculation and miscalculation of the allometric equation as a model in biological data BioScience 1968 18 118 1120

[B116] Warton DI Wright IJ Falster DS Westoby M Bivariate line-fitting methods for allometry Biol Rev 2006 81 259 291 16573844 10.1017/S1464793106007007

[B117] R-Development-Core-Team R: a language and environment for statistical computing 2010 2.12.0 Vienna, Austria: R Foundation for Statistical Computing

[B118] Warton DI Duursma RA Falster DS Taskinen S smatr 3-an R package for estimation and inference about allometric lines Methods Ecol Evol 2011 3 257 259

[B119] Benjamini Y Hochberg Y Controlling the false discovery rate: a practical and powerful approach to multiple testing J R Stat Soc Series B Stat Methodol 1995 57 289 300

[B120] Curran-Everett D Multiple comparisons: philosophies and illustrations Am J Physiol Regul Integr Comp Physiol 2000 279 R1 R8 10896857 10.1152/ajpregu.2000.279.1.R1

[B121] Felsenstein J Phylogenies and the comparative method Am Naturalist 1985 125 1 15 10.1086/284325

[B122] Maddison WP Maddison DR Mesquite: A modular system for evolutionary analysis. Version 2.6 (build 486): mesquiteproject.org 2006

[B123] Bininda-Emonds OR Cardillo M Jones KE MacPhee RDE Beck RMD Grenyer R Price SA Vos RA Gittleman JL Purvis A The delayed rise of present-day mammals Nature 2007 446 507 512 10.1038/nature05634 17392779

[B124] Spinks PQ Shaffer HB Conflicting mitochondrial and nuclear phylogenies for the widely disjunct *Emys *(Testunides: Emydidae) species complex, and what they tell us about biogeography and hybridization Syst Biol 2009 58 1 20 10.1093/sysbio/syp005 20525565

[B125] Townsend TM Larson A Louis E Macey JR Molecular phylogenetics of Squamata: the position of snakes, amphisbaenians, and didamids, and the root of the squamate tree Syst Biol 2004 53 735 757 10.1080/10635150490522340 15545252

[B126] Vidal N Hedges SB The phylogeny of squamate reptiles lizards, snakes, and amphisbaenians) inferred from nine nuclear protein-coding genes C R Biol 2005 328 1000 1008 10.1016/j.crvi.2005.10.001 16286089

[B127] Ast JC Mitochondrial DNA evidence and evolution in Varanoidea (Squamata) Cladistics 2001 17 211 226 10.1006/clad.2001.0169 34911248

[B128] Gaffney ES Meylan PA Benton MJ A phylogeny of turtles The Phylogeny and Classification of Tetrapods 1988 Oxford, UK: Clarendon Press 157 219

[B129] Engstrom TN Shaffer HB McCord WP Multiple data sets, high homoplasy, and the phylogeny of softshell turtles (Testudinea: Trionychidae) Syst Biol 2004 53 693 710 10.1080/10635150490503053 15545250

[B130] Le M Raxworthy CJ McCord WP Mertz L A molecular phylogeny of tortoises (Testudines: Testudinidae) based on mitochondrial and nuclear genes Mol Phylogenet Evol 2006 40 517 531 10.1016/j.ympev.2006.03.003 16678445

[B131] Okajima Y Kumazawa Y Mitochondrial genomes of acrodont lizards: timing of gene rearrangements and phylogenetic and biogeographic implications BMC Evol Biol 2010 10 141 10.1186/1471-2148-10-141 20465814 PMC2889956

[B132] Naro-Maciel E Le M FitzSimmons NN Amato G Evolutionary relationships of marine turtles: a molecular phylogeny based on nuclear and mitochondrial genes Mol Phylogenet Evol 2008 49 659 662 10.1016/j.ympev.2008.08.004 18760371

[B133] Albert EA San Mauro D García-París M Rüber L Zardoya R Effect of taxon sampling on recovering the phylogeny of squamate reptiles based on complete mitochondrial genome and nuclear gene sequence data Gene 2009 441 12 21 10.1016/j.gene.2008.05.014 18639394

[B134] Amer SAM Kumazawa Y Mitochondrial DNA sequences of the Afro-Arabian spiny-tailed lizards (genus *Uromastyx*; family Agamidae): phylogenetic analyses and evolution of gene arrangements Biol J Linnean Soc 2005 85 247 260 10.1111/j.1095-8312.2005.00485.x

[B135] Wiens JJ Brandley MC Reeder TW Why does a trait evolve multiple times with a clade? Repeated evolution of snakelike body form in squamate reptiles Evolution 2006 60 123 141 16568638

[B136] Okajima Y Kumazawa Y Mitogenomic prespectives into iguanid phylogeny and biogeogrpahy: Gondwana vicariance for the origin of Madagascan oplurines Gene 2009 441 28 35 10.1016/j.gene.2008.06.011 18598742

[B137] Roos J Aggarwal RK Janke A Extended mitogenomic phylogenetic analyses yield new insight into crocodylian evolution and their survival of the Cretaceous-Tertiary boundary Mol Phylogenet Evol 2007 45 663 673 10.1016/j.ympev.2007.06.018 17719245

[B138] Near TJ Meylan PA Shaffer HB Assessing concordance of fossil calibration points in molecular clock studies: an example using turtles Am Naturalist 2005 165 137 146 10.1086/427734 15729646

[B139] Garland TJ Harvey PH Ives AR Procedures for the analysis of comparative data using phylogenetically independed contracts Syst Biol 1992 41 18 32

[B140] Paradis E Claude J Strimmer K APE: analuses of phylogenetics and evolution in R language Bioinformatics 2004 20 289 290 10.1093/bioinformatics/btg412 14734327

[B141] Hansen TF Bartoszek K Interpreting the evolutionary regression: the interplay between observational and biological errors in phylogenetic comparative studies Syst Biol 2012 61 3 413 425 10.1093/sysbio/syr122 22213708

[B142] Smith RJ Lead review: estimation of body mass in paleontology J Human Evol 2002 43 271 287 10.1006/jhev.2002.0573

[B143] Martins EP Hansen TF Phylogenies and the comparative method: a general approach to incorporating phylogenetic information into the analysis of interspecific data Am Naturalist 1997 149 647 667

[B144] De Esteban-Trivigno S Köhler M New equations for body mass estimation in bovids: testing some procedures when constructing regression functions Mammalian Biol 2011 76 755 761 10.1016/j.mambio.2011.07.004

[B145] Redelstorff R Sanders PM Long and girdle bone histology of *Stegosaurus*: implications for growth and life history J Vertebrate Paleontol 2009 29 1087 1099 10.1671/039.029.0420

