# Supplementary material for: A universal scaling relationship between body mass and proximal limb bone dimensions in quadrupedal terrestrial tetrapods
Source: BMC Biol. 2012 Jul 10;10:60. doi: 10.1186/1741-7007-10-60 (PMC3403949; doi:10.1186/1741-7007-10-60)
Supplement: Additional file 2 — Table S1. Raw and PIC stylopodial scaling in a subset of the mammalian dataset and non-avian reptiles. Mammalian subset corresponds to all taxa < 168 kg in order to better approximate body mass range in the sample of non-avian reptiles. Standardized Major Axis equation shown in the format y = mx + b (b = 0 in PIC). The particular theoretical scaling model (Sim.) followed by the slope is represented by G, geometric similarity, E, elastic similarity, or S, static similarity. Scaling patterns that fall between models are represented by > or <, and those that do not follow any pattern (that is, above or below all predicted models) are represented by a 0. [file 1741-7007-10-60-S2.DOC]

Table S1. Raw and PIC stylopodial scaling in a subset of the mammalian dataset and non-avian reptiles.

Mammalian subset corresponds to all taxa <168 kg in order to better approximate body mass range in the sample of non-avian reptiles. Standardized Major Axis equation shown in the format *y = mx + b* (*b* = 0 in PIC). The particular theoretical scaling model (Sim.) followed by the slope is represented by G, geometric similarity, E, elastic similarity, or S, static similarity. Scaling patterns that fall between models are represented by > or <, and those that do not follow any pattern (that is, above or below all predicted models) are represented by a 0.

|  |  | Raw Data SMA Results | | | | | | | PIC SMA Results | | | | |
| --- | --- | --- | --- | --- | --- | --- | --- | --- | --- | --- | --- | --- | --- |
| Groups | Analysis | N | *m* | *m* 95% CI | *b* | *b* 95% CI | R^2^ | Sim. | N | *m* | *m* 95% CI | R^2^ | Sim. |
| L_F_ vs. C_F_ | Mammalia | 167 | 0.9878 | 1.0302 to 0.9471 | -0.5366 | -0.4575 to -0.6156 | 0.9251 | G | 45 | 1.1880 | 1.2919 to 1.0923 | 0.9290 | >G,<E |
|  | Reptilia | 46 | 1.1751 | 1.3183 to 1.0473 | -0.8115 | -0.5883 to -1.0346 | 0.8560 | >G,<E | 166 | 1.0640 | 1.1275 to 1.004 | 0.8561 | >G,<E |
| L_H_ vs. C_H_ | Mammalia | 166 | 0.9988 | 1.0414 to 0.9578 | -0.5200 | -0.4437 to -0.5962 | 0.9264 | G | 46 | 1.1853 | 1.2789 to 1.0985 | 0.9388 | >G,<E |
|  | Reptilia | 47 | 1.2190 | 1.3355 to 1.1125 | -0.8536 | -0.6723 to -1.0347 | 0.9072 | >G,<E | 165 | 1.0613 | 1.1198 to 1.0058 | 0.8782 | >G,<E |
| L_F_ vs. BM | Mammalia | 167 | 2.8627 | 2.9915 to 2.7393 | -2.1177 | -1.8776 to -2.3576 | 0.9178 | 0 | 45 | 3.5139 | 3.9389 to 3.1348 | 0.8621 | >G,<E |
|  | Reptilia | 46 | 3.2500 | 3.7486 to 2.8177 | -2.4800 | -1.7131 to -3.2468 | 0.7778 | G | 166 | 3.0857 | 3.2694 to 2.9123 | 0.8579 | G |
| C_F_ vs. BM | Mammalia | 174 | 2.8986 | 2.9708 to 2.828 | -0.5634 | -0.4651 to -0.6616 | 0.9732 | <G,>E | 46 | 2.9493 | 3.2232 to 2.6986 | 0.9123 | G |
|  | Reptilia | 47 | 2.7943 | 2.9801 to 2.62 | -0.2653 | -0.057 to -0.4735 | 0.9540 | E | 173 | 2.9032 | 3.0359 to 2.7762 | 0.9115 | G |
| L_H_ vs. BM | Mammalia | 166 | 2.7029 | 2.8061 to 2.6034 | -1.5895 | -1.4045 to -1.7743 | 0.9409 | 0 | 46 | 3.4403 | 3.7883 to 3.1242 | 0.8974 | >G,<E |
|  | Reptilia | 47 | 3.3718 | 3.7039 to 3.0694 | -2.5472 | -2.0315 to -3.0629 | 0.9018 | >G,<E | 165 | 2.9213 | 3.0735 to 2.7765 | 0.8913 | G |
| C_H_ vs. BM | Mammalia | 174 | 2.7044 | 2.7749 to 2.6356 | -0.1780 | -0.0848 to -0.2711 | 0.9707 | E | 46 | 2.9024 | 3.1359 to 2.6862 | 0.9341 | G |
|  | Reptilia | 47 | 2.7661 | 2.9296 to 2.6117 | -0.1862 | -0.0048 to -0.3675 | 0.9634 | E | 173 | 2.7313 | 2.8571 to 2.6109 | 0.9100 | E |
| L_F_ vs. L_H_ | Mammalia | 166 | 1.0585 | 1.0886 to 1.029 | -0.1937 | -0.1369 to -0.2504 | 0.9666 | - | 45 | 0.9956 | 1.0706 to 0.9257 | 0.9427 | - |
|  | Reptilia | 46 | 0.9644 | 1.0644 to 0.8738 | 0.0190 | 0.1759 to -0.1378 | 0.8943 | - | 165 | 1.0562 | 1.095 to 1.0185 | 0.9443 | - |
| C_H+F_ vs. BM | Mammalia | 174 | 2.8203 | 2.879 to 2.76265 | -1.2486 | -1.1526 to -1.3446 | 0.9812 | - | 46 | 2.9357 | 3.1813 to 2.709 | 0.9285 | - |
|  | Reptilia | 47 | 2.7933 | 2.9496 to 2.6452 | -1.0833 | -0.8635 to -1.3031 | 0.9671 | - | 173 | 2.8585 | 2.9714 to 2.7498 | 0.9334 | - |

BM - body mass

L_F_ - femoral length

C_F_ - femoral circumference

L_H_ - humeral length

C_H_ - humeral circumference

C_H+F_ - total humeral and femoral circumference
