# Supplementary material for: A universal scaling relationship between body mass and proximal limb bone dimensions in quadrupedal terrestrial tetrapods
Source: BMC Biol. 2012 Jul 10;10:60. doi: 10.1186/1741-7007-10-60 (PMC3403949; doi:10.1186/1741-7007-10-60)
Supplement: Additional file 3 — Table S2. Phylogenetically corrected stylopodial scaling in mammals and non-avian reptiles. Scaling equation shown in the format y = mx. The particular theoretical scaling model (Sim.) followed by the slope is represented by G, geometric similarity, E, elastic similarity, or S, static similarity. Scaling patterns that fall between models are represented by > or <, and those that do not follow any pattern (that is, above or below all predicted models) are represented by a 0. [file 1741-7007-10-60-S3.DOC]

**Table S2. Phylogenetically corrected stylopodial scaling in mammals and non-avian reptiles.**

Scaling equation shown in the format *y = mx*. The particular theoretical scaling model (Sim.) followed by the slope is represented by G, geometric similarity, E, elastic similarity, or S, static similarity. Scaling patterns that fall between models are represented by > or <, and those that do not follow any pattern (that is, above or below all predicted models) are represented by a 0.

| Analysis  (*x* vs. *y*) | Sample | N | *m* | *m* 95% CI | R^2^ | Sim. |
| --- | --- | --- | --- | --- | --- | --- |
| L_F_ vs. C_F_ | All | 233 | 1.0788 | 1.1258 to 1.0337 | 0.8909 | >G,<E |
|  | Mammalia | 187 | 1.0651 | 1.118 to 1.0148 | 0.8874 | >G,<E |
|  | Reptilia | 45 | 1.1880 | 1.2919 to 1.0924 | 0.9290 | >G,<E |
|  | Ungulata | 31 | 1.1560 | 1.297 to 1.0303 | 0.8979 | >G,<E |
|  | Carnivora | 45 | 0.9350 | 1.0493 to 0.8332 | 0.8553 | G |
|  | Marsupialia | 13 | 1.0832 | 1.193 to 0.9836 | 0.9678 | G |
|  | Euarchonta | 13 | 1.1223 | 1.4793 to 0.8514 | 0.8083 | G,E |
|  | Glires | 65 | 1.1363 | 1.2512 to 1.0318 | 0.8506 | >G,<E |
| L_H_ vs. C_H_ | All | 233 | 1.0973 | 1.1416 to 1.0547 | 0.9074 | >G,<E |
|  | Mammalia | 186 | 1.0855 | 1.1355 to 1.0375 | 0.9037 | >G,<E |
|  | Reptilia | 46 | 1.1853 | 1.2789 to 1.0985 | 0.9388 | >G,<E |
|  | Ungulata | 31 | 1.2592 | 1.3799 to 1.1489 | 0.9395 | >G,<E |
|  | Carnivora | 45 | 1.0412 | 1.1518 to 0.941 | 0.8888 | G |
|  | Marsupialia | 13 | 1.0619 | 1.2379 to 0.9109 | 0.9282 | G |
|  | Euarchonta | 13 | 1.0725 | 1.4129 to 0.814 | 0.8199 | G |
|  | Glires | 65 | 1.0782 | 1.1536 to 1.0076 | 0.9267 | >G,<E |
| L_F_ vs. BM | All | 233 | 3.1327 | 3.2749 to 2.9965 | 0.8828 | >G,<E |
|  | Mammalia | 187 | 3.0855 | 3.2386 to 2.9395 | 0.8882 | G |
|  | Reptilia | 45 | 3.5139 | 3.9389 to 3.1348 | 0.8621 | >G,<E |
|  | Ungulata | 31 | 3.3430 | 3.7769 to 2.9589 | 0.8852 | G |
|  | Carnivora | 45 | 2.8681 | 3.2383 to 2.5401 | 0.8393 | G |
|  | Marsupialia | 13 | 3.4808 | 4.0791 to 2.9701 | 0.9154 | E |
|  | Euarchonta | 13 | 3.3664 | 4.6175 to 2.4542 | 0.7459 | E |
|  | Glires | 65 | 3.1273 | 3.3921 to 2.8831 | 0.8980 | G |
| C_F_ vs. BM | All | 246 | 2.9115 | 3.0128 to 2.8136 | 0.9261 | G |
|  | Mammalia | 199 | 2.9058 | 3.0165 to 2.799 | 0.9287 | G |
|  | Reptilia | 46 | 2.9493 | 3.2232 to 2.6986 | 0.9123 | E |
|  | Ungulata | 40 | 2.9790 | 3.2356 to 2.7427 | 0.9310 | G |
|  | Carnivora | 47 | 3.0589 | 3.3484 to 2.7943 | 0.9068 | G |
|  | Marsupialia | 13 | 3.2133 | 3.6006 to 2.8676 | 0.9554 | G |
|  | Euarchonta | 14 | 2.9518 | 3.2729 to 2.6622 | 0.9701 | G,E |
|  | Glires | 65 | 2.7522 | 2.9952 to 2.5288 | 0.8876 | E |
| L_H_ vs. BM | All | 233 | 3.0135 | 3.1346 to 2.8969 | 0.9077 | G |
|  | Mammalia | 186 | 2.9539 | 3.0838 to 2.8293 | 0.9123 | G |
|  | Reptilia | 46 | 3.4403 | 3.7883 to 3.1242 | 0.8974 | >G,<E |
|  | Ungulata | 31 | 3.0640 | 3.4547 to 2.7174 | 0.8959 | G |
|  | Carnivora | 45 | 2.9738 | 3.2944 to 2.6842 | 0.8857 | G |
|  | Marsupialia | 13 | 3.1335 | 3.7171 to 2.6413 | 0.9016 | G |
|  | Euarchonta | 13 | 2.9668 | 3.8852 to 2.2654 | 0.8292 | G,E |
|  | Glires | 65 | 2.8571 | 3.0576 to 2.6697 | 0.9278 | G |
| C_H_ vs. BM | All | 246 | 2.7254 | 2.818 to 2.6358 | 0.9295 | E |
|  | Mammalia | 199 | 2.6990 | 2.8012 to 2.6004 | 0.9295 | E |
|  | Reptilia | 46 | 2.9024 | 3.1359 to 2.6862 | 0.9341 | G |
|  | Ungulata | 40 | 2.4023 | 2.6051 to 2.2152 | 0.9337 | S |
|  | Carnivora | 47 | 2.8622 | 3.129 to 2.6181 | 0.9096 | G,E |
|  | Marsupialia | 13 | 2.9508 | 3.4882 to 2.4961 | 0.9183 | G,E,S |
|  | Euarchonta | 14 | 2.7329 | 3.0467 to 2.4514 | 0.9670 | G,E,S |
|  | Glires | 65 | 2.6499 | 2.8328 to 2.4787 | 0.9319 | E,S |
| L_F_ vs. L_H_ | All | 232 | 1.0387 | 1.068 to 1.0101 | 0.9537 | - |
|  | Mammalia | 186 | 1.0442 | 1.0766 to 1.0126 | 0.9552 | - |
|  | Reptilia | 45 | 0.9956 | 1.0706 to 0.9257 | 0.9427 | - |
|  | Ungulata | 31 | 1.0911 | 1.1564 to 1.0293 | 0.9764 | - |
|  | Carnivora | 45 | 0.9645 | 1.0235 to 0.9088 | 0.9618 | - |
|  | Marsupialia | 13 | 1.1108 | 1.337 to 0.9228 | 0.8918 | - |
|  | Euarchonta | 13 | 1.1347 | 1.3601 to 0.9466 | 0.9108 | - |
|  | Glires | 65 | 1.0946 | 1.1499 to 1.0418 | 0.9615 | - |
| C_H+F_ vs. BM | All | 246 | 2.8463 | 2.932 to 2.7631 | 0.9444 | - |
|  | Mammalia | 199 | 2.8325 | 2.9249 to 2.7429 | 0.9473 | - |
|  | Reptilia | 46 | 2.9357 | 3.1813 to 2.709 | 0.9285 | - |
|  | Ungulata | 40 | 2.6829 | 2.8825 to 2.497 | 0.9480 | - |
|  | Carnivora | 47 | 2.9730 | 3.2331 to 2.7337 | 0.9199 | - |
|  | Marsupialia | 13 | 3.1220 | 3.4650 to 2.8129 | 0.9629 | - |
|  | Euarchonta | 14 | 2.8459 | 3.1375 to 2.5813 | 0.9734 | - |
|  | Glires | 65 | 2.7539 | 2.9332 to 2.5855 | 0.9395 | - |

BM - body mass

L_F_ - femoral length

C_F_ - femoral circumference

L_H_ - humeral length

C_H_ - humeral circumference

C_H+F_ - total humeral and femoral circumference
