# Supplementary material for: A universal scaling relationship between body mass and proximal limb bone dimensions in quadrupedal terrestrial tetrapods
Source: BMC Biol. 2012 Jul 10;10:60. doi: 10.1186/1741-7007-10-60 (PMC3403949; doi:10.1186/1741-7007-10-60)
Supplement: Additional file 4 — Table S3. Raw and PIC stylopodial scaling in Artiodactyla and Bovidae. Standardized Major Axis equation shown in the format y = mx + b (b = 0 in PIC). The particular theoretical scaling model (Sim.) followed by the slope is represented by G, geometric similarity, E, elastic similarity, or S, static similarity. Scaling patterns that fall between models are represented by > or <, and those that do not follow any pattern (that is, above or below all predicted models) are represented by a 0. [file 1741-7007-10-60-S4.DOC]

**Table S3. Raw and PIC stylopodial scaling in Artiodactyla and Bovidae.**

Standardized Major Axis equation shown in the format *y = mx + b* (*b* = 0 in PIC). The particular theoretical scaling model (Sim.) followed by the slope is represented by G, geometric similarity, E, elastic similarity, or S, static similarity. Scaling patterns that fall between models are represented by > or <, and those that do not follow any pattern (that is, above or below all predicted models) are represented by a 0.

|  |  | Raw Data SMA Results | | | | | | | PIC SMA Results | | | | |
| --- | --- | --- | --- | --- | --- | --- | --- | --- | --- | --- | --- | --- | --- |
| Groups | Analysis | N | *m* | *m* 95% CI | *b* | *b* 95% CI | R^2^ | Sim. | N | *m* | *m* 95% CI | R^2^ | Sim. |
| Bovidae | flfc | 13 | 1.1070 | 1.3723 to 0.8929 | -0.7480 | -0.173 to -1.3229 | 0.8909 | G | 12 | 1.1531 | 1.5416 to 0.8624 | 0.8133 | G,E |
|  | hlhc | 13 | 1.1678 | 1.3857 to 0.984 | -0.8030 | -0.3356 to -1.2703 | 0.8874 | G | 12 | 1.1513 | 1.434 to 0.9243 | 0.9081 | G |
|  | flbm | 13 | 3.2973 | 3.9924 to 2.7232 | -2.9690 | -1.4467 to -4.4912 | 0.9290 | G | 12 | 3.4556 | 4.525 to 2.6388 | 0.8426 | G,E |
|  | fcbm | 20 | 3.0957 | 3.3733 to 2.8409 | -0.9512 | -0.4385 to -1.4638 | 0.8979 | G | 19 | 3.1673 | 3.5813 to 2.8011 | 0.9379 | G |
|  | hlbm | 13 | 3.3338 | 4.0989 to 2.7115 | -2.8181 | -1.2036 to -4.4325 | 0.8553 | G,E | 12 | 3.2715 | 4.3059 to 2.4855 | 0.8444 | G,E |
|  | hcbm | 20 | 2.5940 | 2.8665 to 2.3474 | 0.0122 | 0.5127 to -0.4882 | 0.9678 | E,S | 19 | 2.5269 | 2.8351 to 2.2522 | 0.9456 | E,S |
|  | flhl | 13 | 0.9891 | 1.1017 to 0.8878 | -0.0453 | 0.2112 to -0.3018 | 0.8083 | - | 12 | 1.0563 | 1.1839 to 0.9424 | 0.9715 | - |
|  | hcfcbm | 20 | 2.8489 | 3.076 to 2.6385 | -1.3367 | -0.8494 to -1.8239 | 0.8506 | - | 19 | 2.8455 | 3.1446 to 2.5748 | 0.9590 | - |
| Artiodactyla | flfc | 26 | 1.1409 | 1.2947 to 1.0053 | -0.8399 | -0.4833 to -1.1964 | 0.9074 | >G,<E | 25 | 1.1480 | 1.3096 to 1.0062 | 0.8889 | G |
|  | hlhc | 26 | 1.2526 | 1.3877 to 1.1305 | -1.0152 | -0.7076 to -1.3226 | 0.9037 | >G,<E | 25 | 1.2482 | 1.3858 to 1.1241 | 0.9356 | >G,<E |
|  | flbm | 26 | 3.3611 | 3.8167 to 2.9598 | -3.1304 | -2.0748 to -4.1858 | 0.9388 | >G,<E | 25 | 3.2810 | 3.7657 to 2.8586 | 0.8805 | G |
|  | fcbm | 34 | 2.9937 | 3.2067 to 2.7947 | -0.7410 | -0.336 to -1.146 | 0.9395 | G | 33 | 2.9651 | 3.2428 to 2.7111 | 0.9324 | G |
|  | hlbm | 26 | 3.1906 | 3.5841 to 2.8401 | -2.4783 | -1.5887 to -3.3677 | 0.8888 | G | 25 | 3.0167 | 3.4531 to 2.6353 | 0.8932 | G |
|  | hcbm | 34 | 2.4813 | 2.7106 to 2.2713 | 0.2501 | 0.6838 to -0.1836 | 0.9282 | E,S | 33 | 2.3860 | 2.6049 to 2.1854 | 0.9347 | S |
|  | flhl | 26 | 1.0725 | 1.4129 to 0.814 | -0.2044 | -0.0146 to -0.394 | 0.8199 | - | 25 | 1.0876 | 1.1593 to 1.0203 | 0.9757 | - |
|  | hcfcbm | 34 | 1.0782 | 1.1536 to 1.0076 | -1.0639 | -0.6401 to -1.4876 | 0.9267 | - | 33 | 2.6658 | 2.8816 to 2.4662 | 0.9487 | - |

flfc - femoral length vs. femoral circumference

hlhc - humeral length vs. humeral circumference

flbm - femoral length vs. body mass

fcbm - femoral circumference vs. body mass

hlbm - humeral circumference vs. body mass

hcbm - humeral circumference vs. body mass

flhl - femoral length vs. humerual length

hcfcbm - total humeral and femoral circumference vs. body mass
