# Supplementary material for: A universal scaling relationship between body mass and proximal limb bone dimensions in quadrupedal terrestrial tetrapods
Source: BMC Biol. 2012 Jul 10;10:60. doi: 10.1186/1741-7007-10-60 (PMC3403949; doi:10.1186/1741-7007-10-60)
Supplement: Additional file 5 — Table S4. Predictive power of various body mass estimation equations. Bivariate and multiple regression statistics for various body mass proxies discussed here (that is, circumference and length of the humerus and femur). Statistics include the Percent Prediction Error (PPE), along with its upper and lower 95% PPE Confidence Intervals (PPE CI), the Standard Error of the Estimate (SEE), the Coefficient of Determination (R2), and the Akaike Information Criterion Score (AIC). [file 1741-7007-10-60-S5.DOC]

**Table S4. Predictive power of various body mass estimation equations**.

Bivariate and multiple regression statistics for various body mass proxies discussed here (that is, circumference and length of the humerus and femur). Statistics include the Percent Prediction Error (PPE), along with its upper and lower 95% PPE Confidence Intervals (PPE CI), the Standard Error of the Estimate (SEE), the Coefficient of Determination (R^2^), and the Akaike Information Criterion Score (AIC).

| **Equation** | **PPE** | **PPE CI** | **SEE** | **R^2^** | **AIC** |
| --- | --- | --- | --- | --- | --- |
| **OLS Regression** |  |  |  |  |  |
| 1. logBM = 2.749logC_H+F_ - 1.104 | **25.63** | 28.513 to 22.748 | 0.134 | 0.988 | -281.882 |
| 2. logBM = 2.818logC_F_ - 0.417 | **33.934** | 38.224 to 29.645 | 0.175 | 0.979 | -152.014 |
| 3. logBM = 2.651logC_H_ - 0.089 | **26.922** | 30.117 to 23.727 | 0.143 | 0.986 | -251.996 |
| 4. logBM = 2.843logL_F_ - 2.005 | **70.822** | 87.438 to 54.206 | 0.311 | 0.93 | 122.345 |
| 5. logBM = 2.802logL_H_ - 1.716 | **50.658** | 57.212 to 44.104 | 0.264 | 0.95 | 46.983 |
| **Non-linear Regression** |  |  |  |  |  |
| BM = 0.421C_H+F_^2.47^ | **42.321** | 45.021 to 39.622 | - | - | - |
| **Multiple Regression** |  |  |  |  |  |
| 1. logBM = 0.375logL_H_ + 1.544logC_H_ - 0.136logL_F_ + 0.954logC_F_ - 0.351 | **24.462** | 27.308 to 21.616 | 0.13 | 0.987 | -275.173 |
| 2. logBM = 1.78logC_H_ + 0.939logC_F_ - 0.215 | **24.932** | 27.64 to 22.224 | 0.131 | 0.988 | -293.67 |
| 3. logBM = 2.432logL_H_ + 0.379logL_F_ - 1.763 | **50.263** | 56.719 to 43.807 | 0.262 | 0.951 | 42.203 |
| **Phylogenetic GLS Regression** |  |  |  |  |  |
| 1. logBM = 2.754logC_H+F_ - 1.097 | **25.03** | 27.711 to 22.349 | 0.135 | - | -232 |
| 2. logBM = 0.212logL_H_ + 1.347logC_H_ - 0.533logL_F_ + 0.749logC_F_ – 0.76 | **26.326** | 29.184 to 23.467 | 0.146 | - | -220.396 |
| 3. logBM = 1.54logC_H_ + 1.195logC_F_ - 0.234 | **24.624** | 27.221 to 22.027 | 0.132 | - | -227.056 |

BM - body mass

L_F_ - femoral length

C_F_ - femoral circumference

L_H_ - humeral length

C_H_ - humeral circumference

C_H+F_ - total humeral and femoral circumference
