# Supplementary figures and images for: A universal scaling relationship between body mass and proximal limb bone dimensions in quadrupedal terrestrial tetrapods
Source: BMC Biol. 2012 Jul 10;10:60. doi: 10.1186/1741-7007-10-60 (PMC3403949; doi:10.1186/1741-7007-10-60)

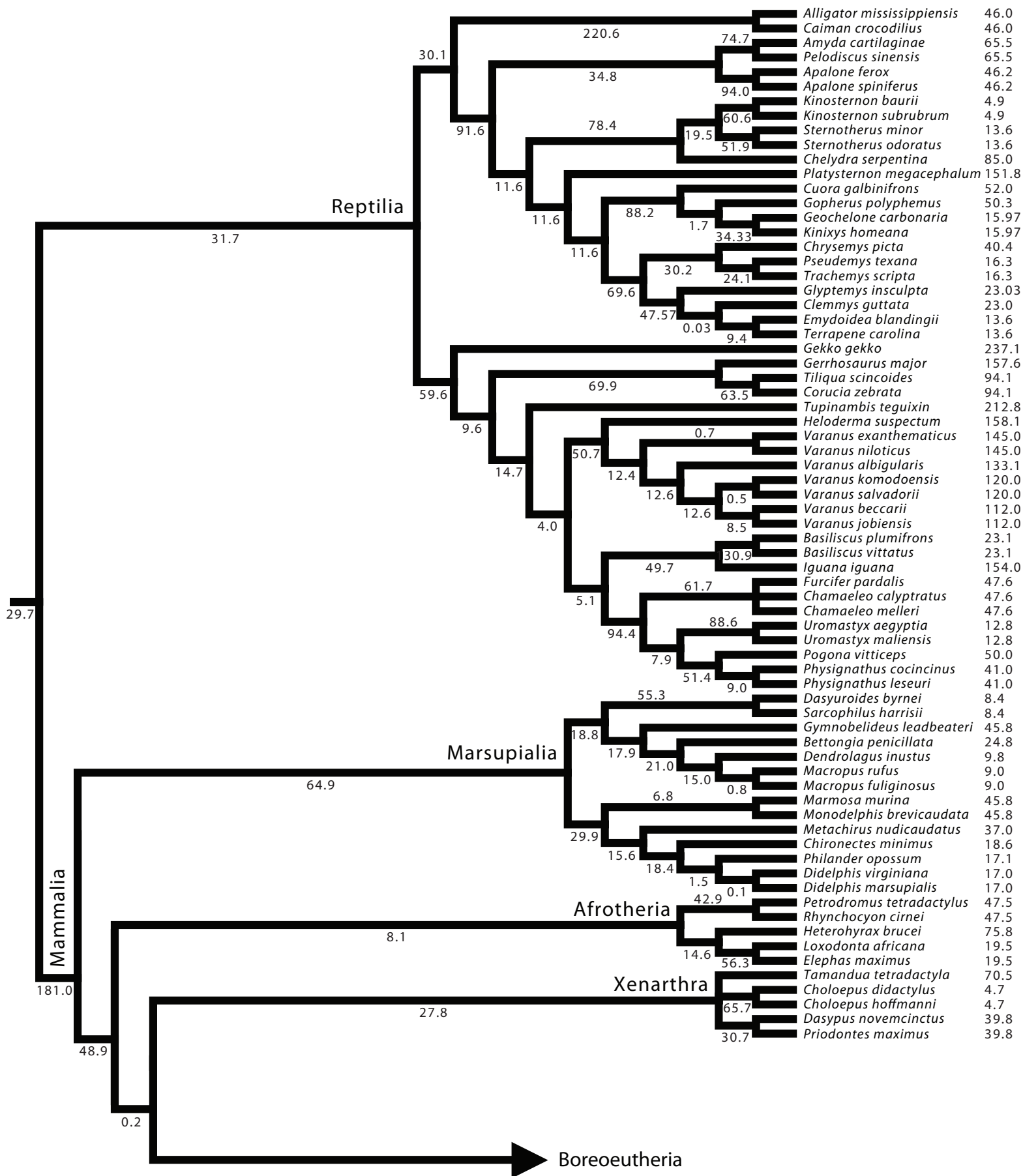

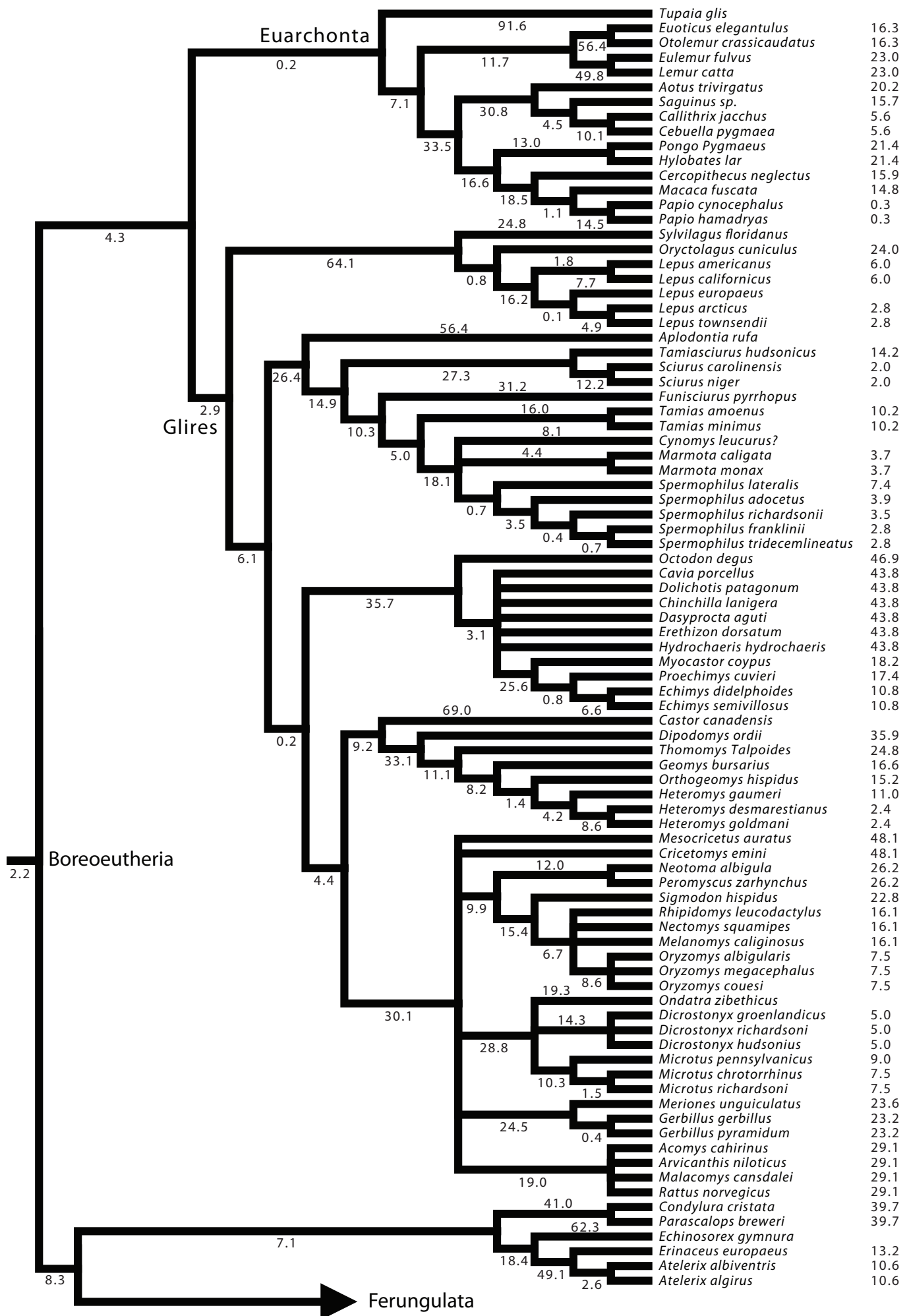

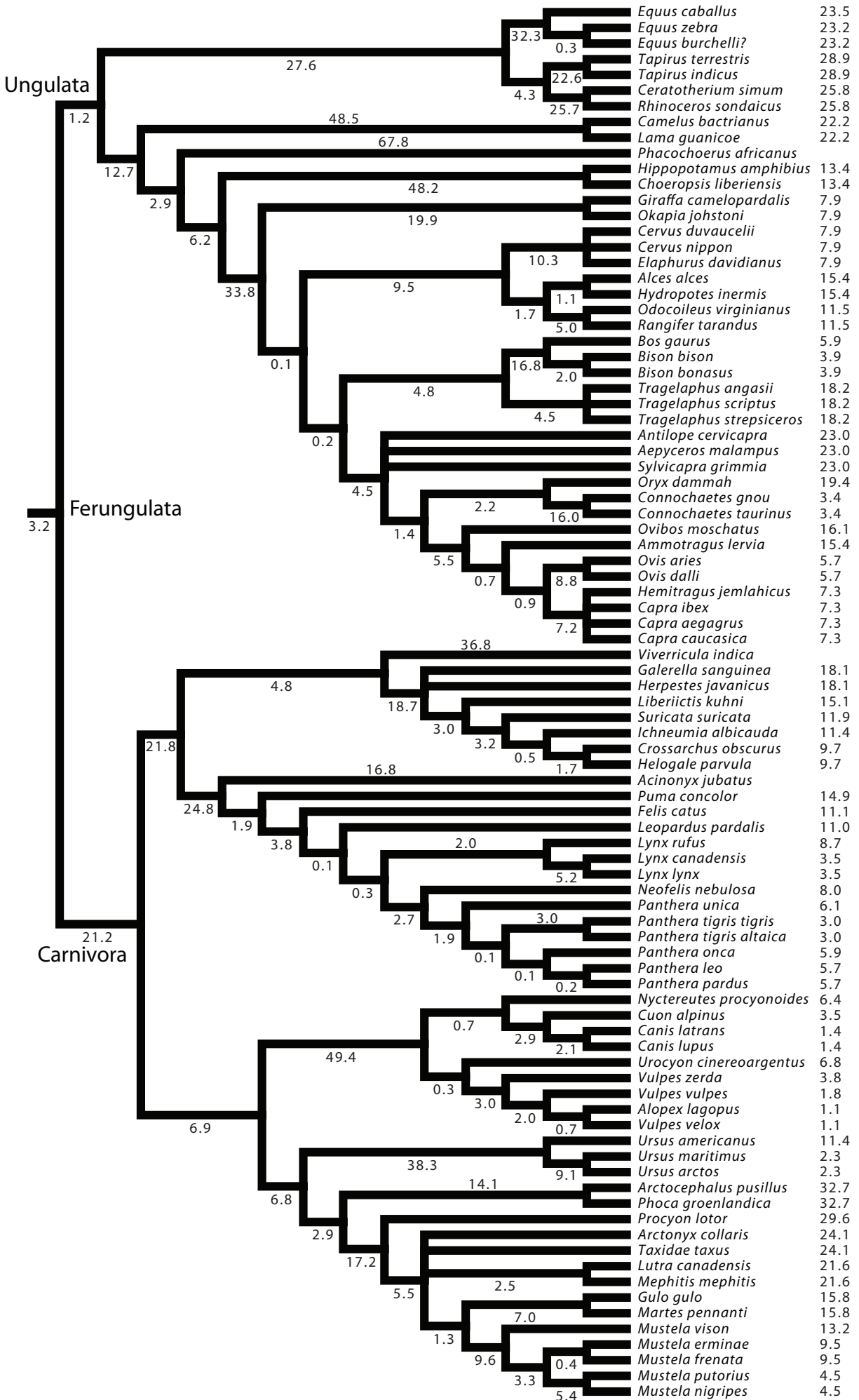

Supplement: Additional file 6 — Figure S1. Phylogenetic tree of mammalian and reptilian taxa included in this study. Topology is based on multiple published analyses mentioned in the text. Numbers indicate the branch lengths used in this study, measured in millions of years. Terminal branch lengths are most often given next to the species name. [file 1741-7007-10-60-S6.PDF]
